# Synergistic graphene–MXene heterostructures for enhanced energy storage in supercapacitors

**DOI:** 10.1039/d6ra07651c

**Published:** 2026-09-29

**Authors:** Sakhi Ghulam Sarwar, Muhammad Ahsan Iqbal, Naveed Zafar Ali, Muhammad Arshad, Usama Shabbir, Mohammed Ashraf Gondal, Qurat-ul Ain, Shahzad Naseem, Serdar Altin

**Affiliations:** a Centre of Excellence in Solid-State Physics, University of the Punjab Lahore Pakistan; b CoE-Physics, National Centre for Physics, Quaid-i-Azam University Campus Islamabad 44000 Pakistan; c School of Materials Science and Engineering, Beijing Institute of Technology Beijing China; d Department of Physics, Division of Science and Technology, University of Education Lahore 54770 Pakistan; e Nanosciences and Technology Department, National Centre for Physics, Quaid-i-Azam University Campus Islamabad 44000 Pakistan arshad_pr2002@yahoo.com +92-3325084848; f Department of Physics, Quaid-i-Azam University Islamabad 45320 Pakistan; g Laser Research Group, Department of Physics & Interdisciplinary Research Center for Hydrogen Technologies and Carbon Management, King Fahd University of Petroleum and Minerals (KFUPM) Dhahran 31261 Saudi Arabia; h Battery Technology Application and Research Center & Physics Department, Inonu University 44280 Malatya Türkiye serdar.altin@inonu.edu.tr +90-536-9664468; i Inonu University, Physics Department 44280 Malatya Türkiye

## Abstract

The restacking of Ti_3_C_2_T_*x*_ MXene nanosheets prevents ions from being accessible and restricts the practical charge-storage performance of MXene-based supercapacitor electrodes. This study aims to explore the growth of MXene (Ti_3_C_2_T_*x*_)/graphene composites with controlled graphene contents, synthesized using a simple one-step ultrasonication method. X-ray diffraction (XRD), field emission scanning electron microscopy (FESEM), Raman spectroscopy and Fourier transform infrared spectroscopy (FTIR) were performed to characterize the as-synthesized MXene/graphene composites and ascertain the successful incorporation of graphene-like honeycomb flakes into the Ti_3_C_2_T_*x*_ sheets. The electrochemical evaluation of fabricated samples was investigated using galvanostatic charge–discharge (GCD), electrochemical impedance spectroscopy (EIS), and cyclic voltammetry (CV) techniques. Among all the fabricated electrodes, the MG-II composites with 30% filler concentration within the MXene sandwich structure demonstrated a greater specific capacitance of 366.3 F g^−1^ when subjected to a current density of 0.5 A g^−1^. This electrode material also exhibited satisfactory cycling stability, with approximately 85.39% retention following 5000 cycles at 1 A g^−1^ with a coulombic efficiency value of 91.69%. In comparison to pristine graphene and Ti_3_C_2_T_*x*_ materials, the as-synthesized composites have shown increased specific capacitance, cycle stability, capacitance retention, coulombic efficiency, and the synergistic interfacial effects of both the two-dimensional structures. Additionally, in the assembly of an asymmetric supercapacitor (ASC), MG-II is utilized as the cathode and activated carbon as the anode. The manufactured device achieved an impressive energy density of 41.95 Wh kg^−1^, leading to a power density of 1000 W kg^−1^, and exhibited 86.6% capacity retention following 10 000 cycles. First-principles density functional theory (DFT) computations revealed enhanced charge mobility through a combination of conduction and valence bands. Analysis of the total density of states (TDOS) and orbital contributions indicated that the Ti_3_C_2_T_*x*_/graphene composite enhances the density of states at the Fermi level, facilitating significant charge transfer from Ti_3_C_2_T_*x*_ to graphene, suggesting improved electronic properties. These results highlight that MXene/graphene shows promise as a reliable, high-performing electrode material for advanced energy storage applications.

## Abstract 2

Ti_3_C_2_T_*x*_ MXene/graphene heterostructures with optimized 30 wt% graphene loading (MG-II) deliver 366.3 F g^−1^ at 0.5 A g^−1^ and 85.39% capacitance retention after 5000 cycles, enabling an asymmetric supercapacitor with 41.95 Wh kg^−1^ energy density.

## Graphical abstract

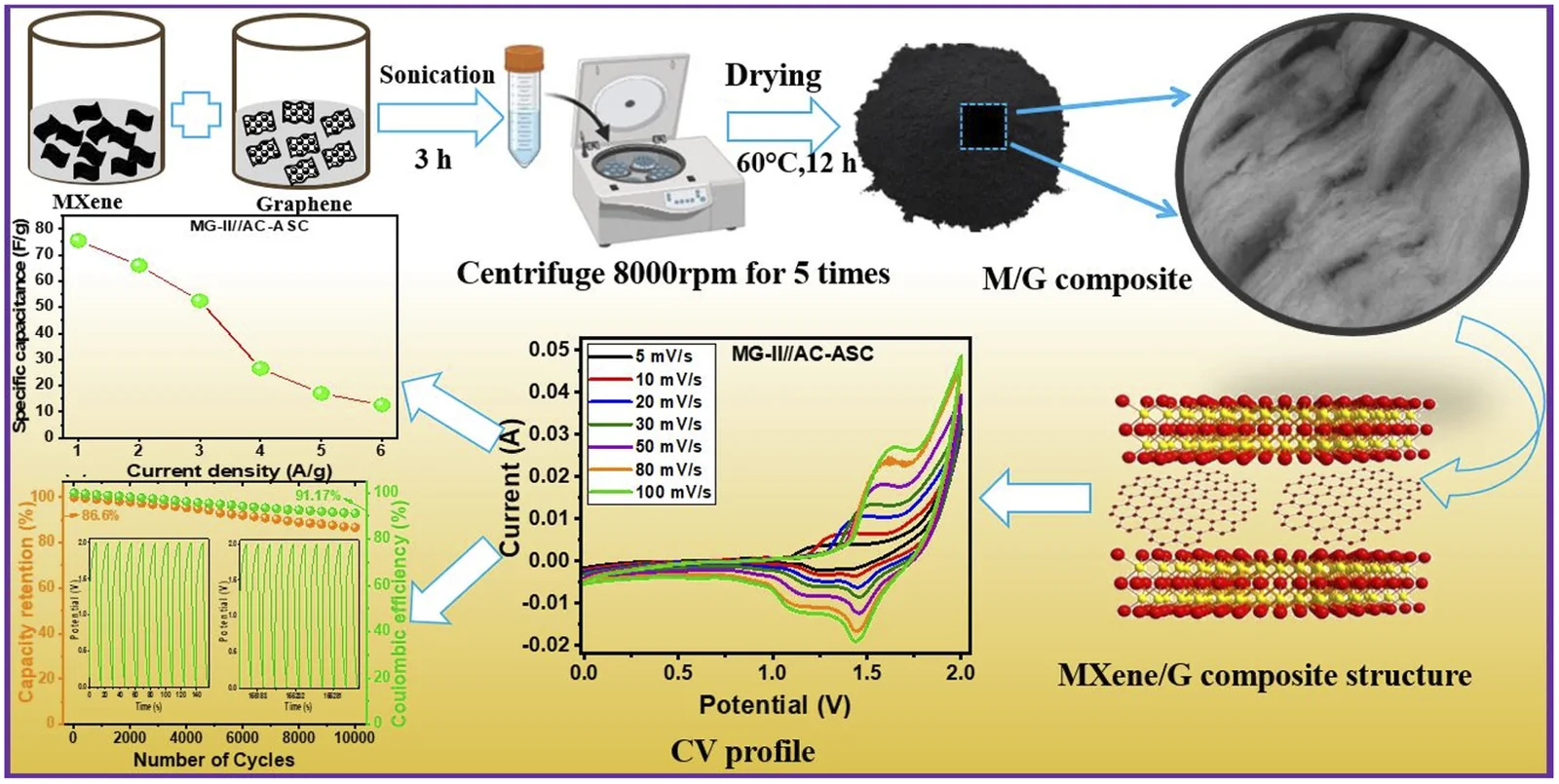

## 1. Introduction

Eco-friendly, renewable energy alternatives have attracted growing interest in mitigating the depletion of traditional energy sources and climatic change. The persistent need for renewable energy sources with no carbon emissions has ignited an increase in the investigation of innovative materials and technologies for energy storage. The increasing reliance on electronic devices underscores the need for novel and efficient energy conservation and transformation systems to function as power sources.^1^ A wide range of materials are undergoing thorough research and application in energy storage systems. These systems, including batteries, fuel cells and solar/wind technologies,^2^*etc.* play a vital role in maximizing the effectiveness of alternative energy sources and ensuring the smooth and eco-friendly functioning of energy-reliant technologies such as electric vehicles and electronic gadgets.^3–6^ Supercapacitors (SCs) are garnering considerable attention as premier energy storage devices owing to their remarkable capacitance, high power density, prolonged lifespan (up to 100 000 cycles),^7^ and economical upkeep.^8,9^ These characteristics render supercapacitors highly suitable for use in high-powered electronic gadgets, backup power sources, and hybrid electric cars. The supercapacitor (SC), which offers the quickest charging and discharging of any electrochemical system and is fit for the fast-paced modern world, has become a key factor in meeting this worldwide requirement.^10^ Constructing electrode assemblies that are both structurally stable and have high surface areas with exceptional mechanical flexibility is a major challenge in SC research.^5,11^ One further major obstacle is making electrochemical capacitors with a higher energy density.^12^ Scholars are furthermore exploring redox-battery type materials revealing pseudocapacitance such as transition metal oxides (TMOs), metal–organic frameworks and transition metal chalcogenide nanostructures.^13^

TMOs have strong pseudocapacitive performance due to their redox properties. As an example, solvothermal-synthesized NiFe_2_O_4_ nanoparticles demonstrate 478.0 F g^−1^ using 1 A g^−1^ and retain 88% cycling stability following 10 000 cycles.^14^ Nanosheets made of Co_3_O_4_@MnO_2_, resembling leaves, exhibit impressive electrochemical features, demonstrating a capacitance value of 616.7 F g^−1^ at a rate of 2 A g^−1^ and strong resistance to cyclic degradation, retaining 83.1% of initial value after 10 000 cycles at 20 A g^−1^.^15^ These illustrations elucidate the manner in which transition metal oxides can construct high-efficiency supercapacitors. However, a lower energy density and restricted electrochemical kinetics remain a major issues.^16^ Instead, this research's MXene/graphene composites are competitive in the energy storage industry due to their elevated conductivity, structural stability, and easy manufacture.

Recently, MXenes have surfaced as an innovative category of two-dimensional compounds composed of transition metal carbides and nitrides with formulation of M(*n* + 1)X(*n*)T(*x*) which the M is a transition metal, X correspond to carbon or nitrogen, T_*x*_ is the functional groups on the surface, and *n* can take the values of 1, 2, or 3. The fabrication of MXenes often entails the extraction of certain layers of A-group elements from MAX phase precursors (M_*n*+1_AX_*n*_) using a method referred to as selective etching.^17^ Since its unveiling in 2011, MXenes have drawn the interest of the scientific community for a range of applications, encompassing supercapacitors,^18^ lithium-ion and sodium-ion batteries, as well as electromagnetic interference shielding,^19^ due to their improved electrochemical performance, high specific capacitance, outstanding hydrophilicity and conductivity, *etc.* MXenes show potential as phase transition materials for energy storage and conversion systems that leverage multiple energy sources, as shown by recent research. For instance, MXene-based phase transition materials control heat well. These materials improve energy storage and conversion.^20^ These findings underscore the significance of interlayer manipulation and composite formulation in enhancing MXene-derived materials for future energy storage applications. Furthermore, graphene is a remarkable two-dimensional carbon-based substance consisting of just a single atom thick layer, has captured global attention in recent decades attributed to its high conductivity, large specific surface area and impressive mechanical characteristics make it a good fit for next-generation smart and portable technologies with improved functionality. Its function as an electrode substance for supercapacitors has been extensively recognized across several sectors and academic disciplines. Sethi *et al.* outlined the eco-friendly fabrication of porous graphene, which enhanced supercapacitor electrode energy density and cycling stability. The energy density of electrodes based on graphene is 85.6 Wh kg^−1^ at room temperature and 136 Wh kg^−1^ at 80 °C which indicates the potential for energy storage devices.^21,22^ The surface area of graphene reaches a staggering 2630 m^2^ g^−1^, highlighting its immense potential for diverse applications beyond conventional materials. This quantity of values is advantageous for carbon-based supercapacitor electrode materials, exceeding both graphite (about 10 m^2^ g^−1^) and CNTs (1315 m^2^ g^−1^).^23^ Solvothermal graphene-NiO nanocomposites demonstrated capacity of 448 F g^−1^ with outstanding capacity retention after 10 000 cycles.^24^ Graphene-NiCo_2_O_4_ nanorod hybrids demonstrated significant energy density with 1027 F g^−1^ capacitance at 1 A g^−1^.^25^ In addition, graphene-NiFe_2_O_4_ composites showed 1051 F g^−1^ capacitance and 91.5% retention after 5000 cycles, demonstrating its importance in enhancing stability and performance.^26^ One can anticipate better results when high electrical conductivity graphene is carefully sliced between MXene nanosheets to greatly increase their surface area and the capacity to prevent both graphene and MXene from self-restacking by creating a structure with alternating MXene/graphene layers.^27^

These concerns highlight the requirement to control MXene interlayer design while retaining accessible conductive pathways. Merging MXene with graphene suggest a practical method to address restacking and transport restrictions, but the optimal graphene content and the related structural, interfacial and charge-storage mechanisms need systematic evaluation. In this work, we have employed the synthesized transition metal carbide MXene (Ti_3_C_2_T_*x*_) and then prepared composites with graphene to investigate their role as electrode materials in supercapacitors. In contrast with previous MXene/graphene studies, which mainly focus on the formation of binary composites, the present study reveals the optimal ratio of graphene content and correlates the electrochemical response with structural regulation, EIS derived charge transfer characteristics, Trasatti/Dunn capacitive contributions, and DFT supported electronic redistribution. The as fabricated supercapacitor electrodes material in addition offers exceptional charge storage capabilities with high structural and cyclic stability.

## 2. Experimental section

### 2.1. Synthesis of Ti_3_C_2_T_*x*_ MXene and graphene

Ti_3_AlC_2_ (>98 wt% purity, Sigma-Aldrich) were used as a precursor. MXene was synthesized using the HF etching, where 20 mL of solution was used with 1 g of T_3_AlC_2_. 12.5 mL of 48% Concentrated hydrofluoric acid was mixed with 7.5 mL of deionized water and placed on a Teflon bar composed of polytetrafluoroethylene on a magnetic heating plate. Then, over a duration of five minutes, the T_3_AlC_2_ MAX phase was gradually introduced at room temperature while being constantly stirred to avoid any exothermic reactions. After sealing the PTFE vessel of the Teflon bar, the temperature was incrementally increased to 50 °C while being constantly stirred, and it was maintained there for six hours. Following centrifugation of the resulting solution at 7000 rpm at ambient temperature, the resulting suspension was centrifuged and washed repeatedly with deionized water with ethanol until the pH reached 6.6. The resultant sediment was moved to a vacuum oven and dried for 24 hours at 60 °C, and prepared for the next intercalation. To minimize oxidation, air exposure was limited throughout the process by carrying out centrifugation and washing promptly, and the dried MXene powder was stored in a sealed vial under Ar atmosphere at low temperature and in the dark, and used for composite and electrode fabrication within a short period, consistent with established handling guidelines for Ti_3_C_2_T_*x*_ MXene.^28^ Furthermore, graphene was synthesized electrochemically by exfoliating a graphite rod functioning as an electrode, in conjunction with a grounded platinum wire. The 0.5 M ammonium persulfate was used as electrolyte. The exfoliation process started by connecting the electrodes to a voltage supply with the graphite rod exfoliating at +10.2 V and reaching complete exfoliation at +12.4 V after 2 to 3 hours. The resulting graphene was washed, collected, and distributed in a DMSO solution to isolate graphite flakes and graphene sheets, thereafter dried at 80 °C for 4 hours.^29^

### 2.2. Synthesis of Ti_3_C_2_ MXene/graphene composite

Ti_3_C_2_T_*x*_/graphene composites were prepared with varying MXene to graphene mass ratios of 85 : 15, 70 : 30, and 55 : 45, labelled as MG-I, MG-II and MG-III by simple ultrasonication method. This composition range (15–45 wt% graphene) was selected because low graphene loadings (below 25 wt%) typically show weaker and less stable synergistic effects on structural and electrical properties in MXene/graphene composites.^30^ As depicted in Fig. 1, the initial step involved dispersing 85 mg of Ti_3_C_2_T_*x*_ powder in 10 mL of NMP and subjecting them to ultrasonic treatment for 2 hours, thus resulting in a homogeneous mixture. Likewise, 15 mg of graphene nanosheets was introduced into a separate beaker containing 10 mL of NMP and ultrasonicated for 2 hours to achieve a well-dispersed state. Subsequently, the graphene dispersion was combined with the Ti_3_C_2_T_*x*_ dispersion, and this Ti_3_C_2_T_*x*_/graphene dispersion was obtained by subjecting it to sonication for another 3 hours. After that, the suspension was centrifuged at 10 000 rpm. Then washed 3–4 times with deionized water to eradicate the contaminants. The solution was dried at 80 °C for 4 hours in a vacuum oven. Similarly, the same method was used to prepare the other weight ratios of composites MG-II and MG-III. This Ti_3_C_2_T_*x*_/graphene composite was then used to construct the electrodes for supercapacitors.

**Fig. 1:**
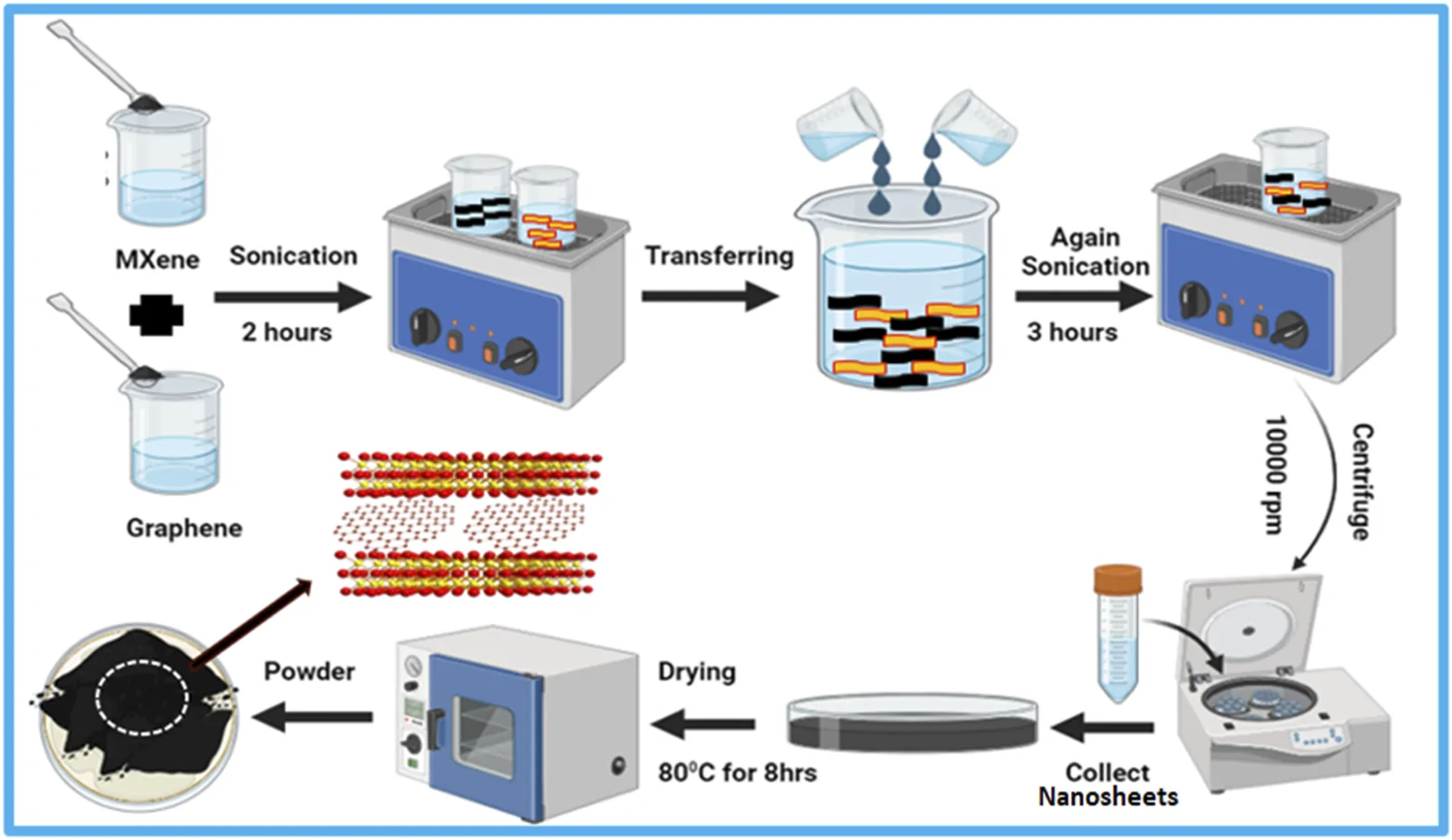
Visual depiction for the creation of MXene/graphene composite.

### 2.3. Characterizations

The synthesized MXene, graphene, and composite materials were characterized by collecting data in the range of 5° ≤ 2*θ* ≤ 80°, with a 2 second integration time for each 0.02° step, using a powder X-ray diffraction (XRD) device (D8 Advance, Bruker) that utilizes Cu Kα radiation (*λ* = 1.5409 Å). Structural refinements were performed with TOPAS software (version 4.1) through the Rietveld refinement method. Given the small crystalline coherence length and turbostratic stacking of Ti_3_C_2_T_*x*_ MXene, the Rietveld refinement was restricted to average structural parameters (lattice parameters and interplanar spacings) and relative phase weight fractions, rather than detailed atomic positions, consistent with the reported limitations of Bragg-based refinement for such disordered layered structures.^31^ The samples' shape and morphological features were examined using Carl Zeiss Field Emission Scanning electron microscopy (FESEM) functioning at an increasing voltage of 10 kV by nanocomposite on a silicon wafer substrate. The dimensions and form of the graphene based MXene composite were analyzed using a JEOL JEM2100F high-resolution transmission electron microscopy (HRTEM) system operated at an accelerating voltage of 200 kV. For the HRTEM examination, the sample solution was created by dispersing the powdered specimen in water, thereafter undergoing ultrasonic treatment for 5 minutes. A 5 µL droplet of the diluted solution was introduced onto a 400-mesh pure carbon grid and subsequently subjected to vacuum drying. Raman spectra were acquired with a Raman spectrometer (Renishaw in Via Reflex with 633 nm laser excitation) to determine the vibrational characteristics. Fourier Transform Infrared Spectroscopy (FTIR) 7800A is utilized to determine the presence of chemical functionalities. The chemical composition of MXene/graphene composites was analyzed using X-ray photoelectron spectroscopy (XPS) with a Scienta-Omicron system from Germany. The X-ray source (Al Kα, *hν* = 1486.7 eV) operated at 270 W. A survey scan was conducted at a pass energy of 100 eV, while detailed scans were performed at 20 eV. Data collection was executed *via* Matrix software, while analysis was performed with Igor Pro, applying XPS fitting methodologies. Comprehensive scans were adjusted to a 70 : 30 Gaussian–Lorentzian shape line profile subsequent to Shirley background adjustments. The system was calibrated with an Au 4f core level at 84.0 eV, with binding energies were referenced to the C 1s peak at 284.8 eV.

### 2.4. Electrochemical measurement

All electrochemical tests were carried out at room temperature in an aqueous 1M KOH electrolyte solution using a Gamry setup to investigation of the electrochemical characteristics of the Ti_3_C_2_T_*x*_/graphene sample. The configuration was employed to investigate the electrochemical attributes of the electrode, proficiently recognizing the redox reaction peaks of molecular species in a three-electrode setup that included graphene, MXene, and their composite as the working electrode, Ag/AgCl as the reference electrode, and platinum wire as the counter electrode. A homogeneous mixture was formed by amalgamating acetylene black, polyvinylidene fluoride (PVDF), and active material in a fixed ratio of 1 : 1 : 8, which was subsequently dried at 60 °C overnight after being applied to (1 × 1) cm nickel foam for the assessment of cyclic voltammetry (CV), EIS, and galvanostatic charge–discharge profiles.

For the two-electrode study, the MG-II is used as cathode and activated carbon as anode to formulate an ASC device. When constructing an ASC, the charge equilibrium for a supercapacitor must conform to the subsequent eq.: *q*_+_ = *q*_−_, the charge retained by each electrode is contingent upon the voltage range during the charging/discharging process (Δ*V*), the specific capacitance (*C*), and the mass of the electrode (*m*) as expressed in the eq. *q* = *C* × Δ*V* × *m*. As a result, the eq. for mass balancing is *m*_+_/*m*_−_ = (*C*_−_ × Δ*V*_−_)/(*C*_+_ × Δ*V*_+_). Therefore, it was determined that the loading mass ratio of active materials (AC/MG-II) was 0.66. The loading mass of active ingredients for this study is 1 mg for AC and 1.5 mg for MG-II.

The specific capacitance, energy density, and power density of the electrode, derived from the GCD, were calculated using the following equations, respectively.^32^1
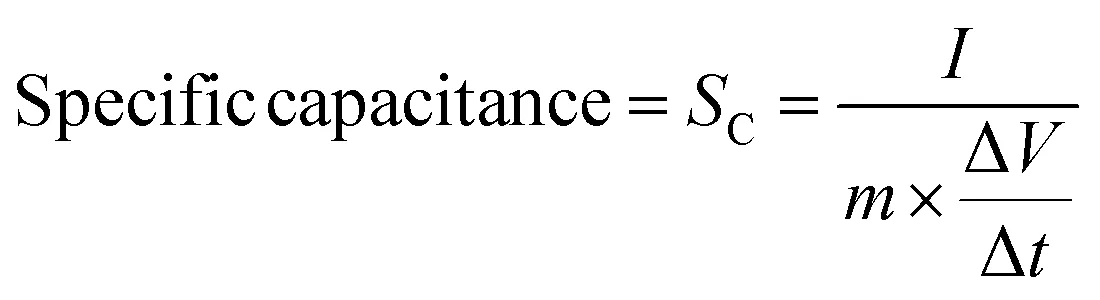
2
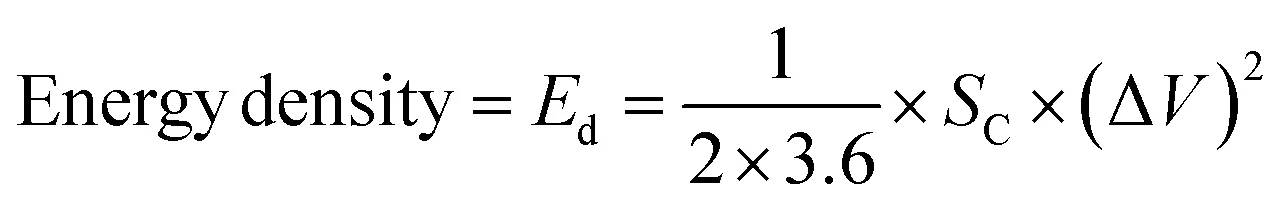
3
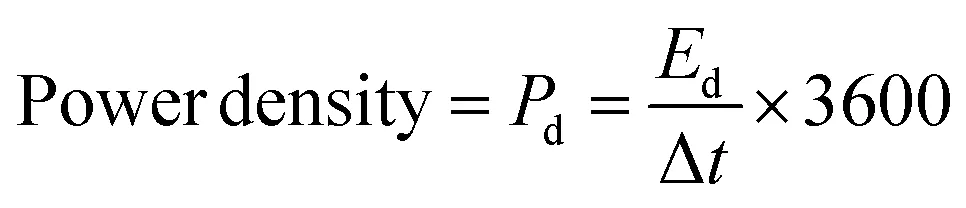
Here, “*S*_C_” signifies the specific capacitances (F g^−1^), “*I*” stands for the current (A), “Δ*t*” represents the discharge duration (s), and “*m*” denotes the mass of the active substance (g), whereas “Δ*V*” relates to the potential range, “*E*_d_” is the energy density quantified in Wh kg^−1^, and “*P*_d_” is the power density defined in kW kg^−1^.

### 2.5. Computational method

We perform structural and electrical optimization tasks using first-principles Density Functional Theory (DFT) simulations with the Vienna *ab initio* simulation package (VASP)^33^ and the projector augmented wave (PAW) approach.^34^ In these simulations, the electron–electron exchange interactions were included with the generalized gradient method of the Perdew–Burke–Ernzerhof functional.^35^ The structural relaxation was conducted by the conjugate gradient approach until the forces acting on the atoms in the structure diminished to levels below 10–4 (eV Å^−1^). We selected an energy cutoff of 400 eV and utilized a Monkhorst–Pack *K*-point grid of 13 × 13 × 7 for the bulk and composite phases.

## 3. Results and discussion

### 3.1. Phase and structure analysis

Fig. 2(a–c) illustrates the outcomes of the estimated Rietveld refinement fitting of pristine MAX, MXene phase and MG-II composite of Ti_3_C_2_T_*x*_/graphene samples. Fig. S1 portrays the XRD pattern of MAX Phase, MXene, graphene and their composites. Fig. S2 contains corresponding quantitative Rietveld refinement analysis of graphene, MG-I (85 : 15), and MG-III (55 : 45) composites in agreement with the added filler concentrations. The crystal structure and lattice parameters obtained through Rietveld fitting for each composition are consolidated in Table S1. The qualitative Rietveld refinement of unaltered PXRD data for Ti_3_AlC_2_ demonstrated a strong correlation with the simulated model, which crystallizes in a hexagonal structure inside the space group *P*6_3_/*mmc*, with lattice parameters of *a* = *b* = 3.073 Å and *c* = 18.58 Å, respectively. There was no Bragg's reflection commensurate to the trivial TiC impurity phase as shown in Fig. 2(a). All the commensurate reflections occurring at 2*θ* value of 9.5°, 19.0°, 34.0°, 36.8°, 38.8°, 41.9°, 45.1°, 48.5°, and 60.2°, respectively flawlessly attributed to the corresponding (002), (004), (101), (103), (104), (105), (106), (109), and (110) phase reflections of Ti_3_AlC_2_ (JCPDS No. 52-0875).^36,37^ The downshift in certain peak intensity along with structural re-arrangement and crystallinity becomes apparent following treatment of Ti_3_AlC_2_ with HF, as indicated in the associated XRD profile. The resultant etching procedures trigger the extraction of Al layers from Ti_3_AlC_2_ phase with the systematic absence of the specific diffraction peaks at 2*θ* = 36.9°, 38.9°, or 42.0° associated with the hexagonal Ti_3_AlC_2_ intrinsic phase.

**Fig. 2:**
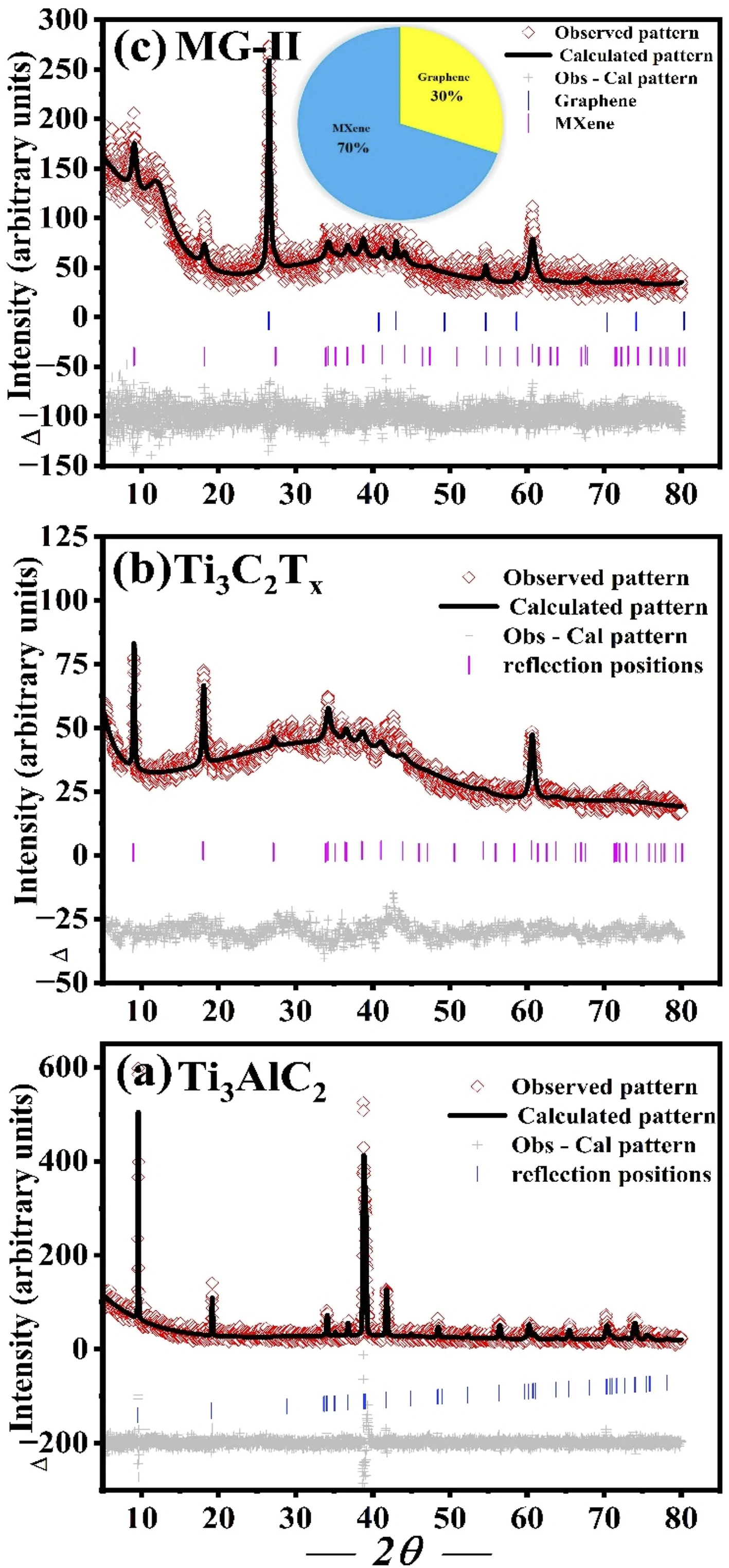
X-ray diffraction along with the Rietveld refinement of (a) Ti_3_AlC_2_, (b) Ti_3_C_2_T_*x*_, and (c) MG-II composite measured at ambient conditions as a function of 2*θ*.

Furthermore, the distinct diffraction peak (104) of Ti_3_AlC_2_ disappeared entirely, suggesting the complete removal of the Al component from the Ti_3_AlC_2_ precursors, while the distinctive (002) peak at 2*θ* = 9.53° broadened and migrated to a reduced angle (2*θ* = 9.00°), indicating an increase in *d*-spacing due to the etching of the Al component with termination groups such as –F, –O, and –OH, along with potential intercalation of ions or molecules.^38^ This selective loss of the (104) reflection, together with the directional shift of the (002) reflection rather than a general redistribution of intensity across multiple reflections, is consistent with Al removal rather than general structural disorder or preferred orientation, as established for the Ti_3_AlC_2_-to-Ti_3_C_2_T_*x*_ transformation.^39,40^ The Lebail refinement of the XRD pattern for pure MXene nanosheets is primarily achieved using *P*6_3_/*mmc* symmetry, with lattice parameters *a* = *b* = 3.033 Å and *c* = 19.365 Å, demonstrating strong concordance between simulated and experimental data, indicated by an *R*_Bragg_ value of 2.336 as seen in Fig. 2(b). The stacking sequence of Ti_3_C_2_T_2_ MXene layers (…L1–L2–L1…) where L2 and L2 represent the two building blocks of the same Ti–C connectivity but with different orientation within the MXene architecture, plays a significant role in evaluating the structural disorder and subsequent broadening of powder XRD intensity in MXene patterns, particularly at higher two theta values (2*θ* = 30° to 50°).

The impact of altering all structural parameters, including atomic positions, composition, site occupancy, stacking vectors, and stacking probability, requires further assessment through Faults simulation and minimization tools to elucidate the intricate structural characteristics of MXene nanosheets, which is the crucial connection in understanding the relationship between functional properties and various synthetic methods and treatments.^41^ The lack of a peak for graphene oxide at 2*θ* ∼14° indicates minimal oxidation in the samples.^42–44^ Whereas in the case of composite samples, all the peaks of MXene and graphene with different weight ratios can be seen with varying intensity as shown in Fig. 2(c), S2(b and c) correspondingly.

In Fig. S2(a), the Rietveld refinement analysis of graphene diffraction patterns reveals a strong correlation between the theoretical and observed patterns, featuring a prominent sharp peak at 26.4° and a lesser broad peak at 43.24°, corresponding to the (002) and (100) graphene planes crystallizing in the space group *P*6_3_/*mmc*. The detection of the (002) plane of graphite in graphene is attributable to the development of many sheets. Through electrochemical exfoliation, ionic substances such as water (H_2_O) and sulphate ions (SO_4_^2−^) are driven to intercalate, resulting in the generation of gas In the realm of MXene materials, the (002) XRD peaks corresponds to the distance between the neighboring MXene layers. A heightened intensity of the (002) peak generally indicates a more meticulously arranged and well-stacked structure. The alteration in the intensity of the (002) peaks in the pure MXene is substantial, yet in the case of the composite, the (002) peaks of MXene are less pronounced due to the addition of varying percentages of graphene. In Fig. S1(c), it can be observed that adding 15% graphene to MXene results in a heightened (002) peaks intensity, signifying that the existence of a small quantity of graphene may encourage a more orderly stacking of MXene layers. This occurrence can be ascribed to the favourable contact and intercalation among the graphene and MXene layers, ultimately enhancing the overall crystal structure of MXene. Conversely, when 30% and 45% of graphene is added to MXene, the smaller intensity of MXene (002) peaks can be seen.^45^ The impact of graphene content on the structure was investigated by performing XRD analysis on MG-I, MG-II, and MG-III composites. As seen in Fig. S1(c), graphene increases the interlayer gap by lowering the (002) peak of MXene. The improved electrochemical performance is result of the facilitated ion transfer made possible by the wider spacing, and the high crystallinity and structural organization brought about by the 30% graphene in the MG-II composite.^46,47^ The wt% Rietveld reveals that in the MG-I sample, the graphene ratio amounts to around 15% with 85% of MXene contents. Whereas the filler ratio increases to 30% in MG-II and heightens up to 45% in MG-III composite samples. There was no impurity phase detected in all these patterns showing the successful incorporation of graphene sheets into the MXene layers. As a result, an increased number of graphene layers intercalate within the MXene layers, thus enhancing the surface area and spacing of MXene layers while inhibiting the aggregation process. This creates a greater number of active sites for charge retention, while the layering facilitates better ion access to the electrode surface. The expected rise in crystallinity of Ti_3_C_2_T_*x*_/graphene. Composite is believed to be responsible for the enhanced electrochemical performance of the as-synthesized electrode materials. The quality of refinements is also assessed by minimal residual *R*-factor (*R*_wp_, *R*_exp_, and *R*_bragg_) values presented in Table S1.

### 3.2 Morphological analysis

Fig. 3(a–c) shows the HR-TEM images of MG-II composite. The thinner regions in the image likely represent exfoliated or delaminated sheets with reduced electron density and darker area indicates the overlapping of layers. The irregular contrast and texture indicate surface imperfections that contribute to increased electrochemical activity by boosting active sites and facilitating ion transport. The results from the selected area electron diffraction (SAED) analysis revealed a distinct diffraction pattern. The main lattice fringes correspond to crystal planes (002), (004), and (103), also consistent with the XRD patterns. Fig. 3(d and e) exhibits the surface morphology of the MXene, and MG-II composites, which were synthesized on a silicon wafer substrate. Shown in Fig. S3(a) illustrates the surface morphological composition of graphene presenting a platelets-like shape. Additionally, clusters of nanosheets can be observed as a result of their expansive surface area. The layered structure of stacked graphene showcases organized designs or crease formations, thereby revealing its potential for energy storage.^48^ The existence of the Al layer in the precursor results in Ti_3_AlC_2_ having a block structure characterized by a smooth surface. The Ti_3_C_2_T_*x*_ acquired through HF etching displays a characteristic sheet structure resembling that of an accordion-like, and the desirable multilayer structure in Fig. 3(d) demonstrates the effective removal of the aluminum atomic layer through selective etching.^49^ The accordion-shaped structure of MXene is believed to be a result of a higher concentration of released gases, such as H_2_, as a result of the exothermic interaction between HF and Ti_3_AlC_2_, while the predetermined etching path of “Al” results in a neighboring arrangement of MXene sheets.^50^ Fig. S3(c) depicts MG-I composite typically reveals a microstructure characterized by a moderate dispersion of graphene sheets within the MXene layers. This represented a well-distributed arrangement of graphene layers integrated between the MXene flakes, thereby illustrating a composite material that possesses improved interfacial interactions between its constituent components. In contrast, the SEM image in Fig. 3(e) of the MG-II composite shows more clearly the presence of uniformly integrated graphene sheets in the MXene structure.

**Fig. 3:**
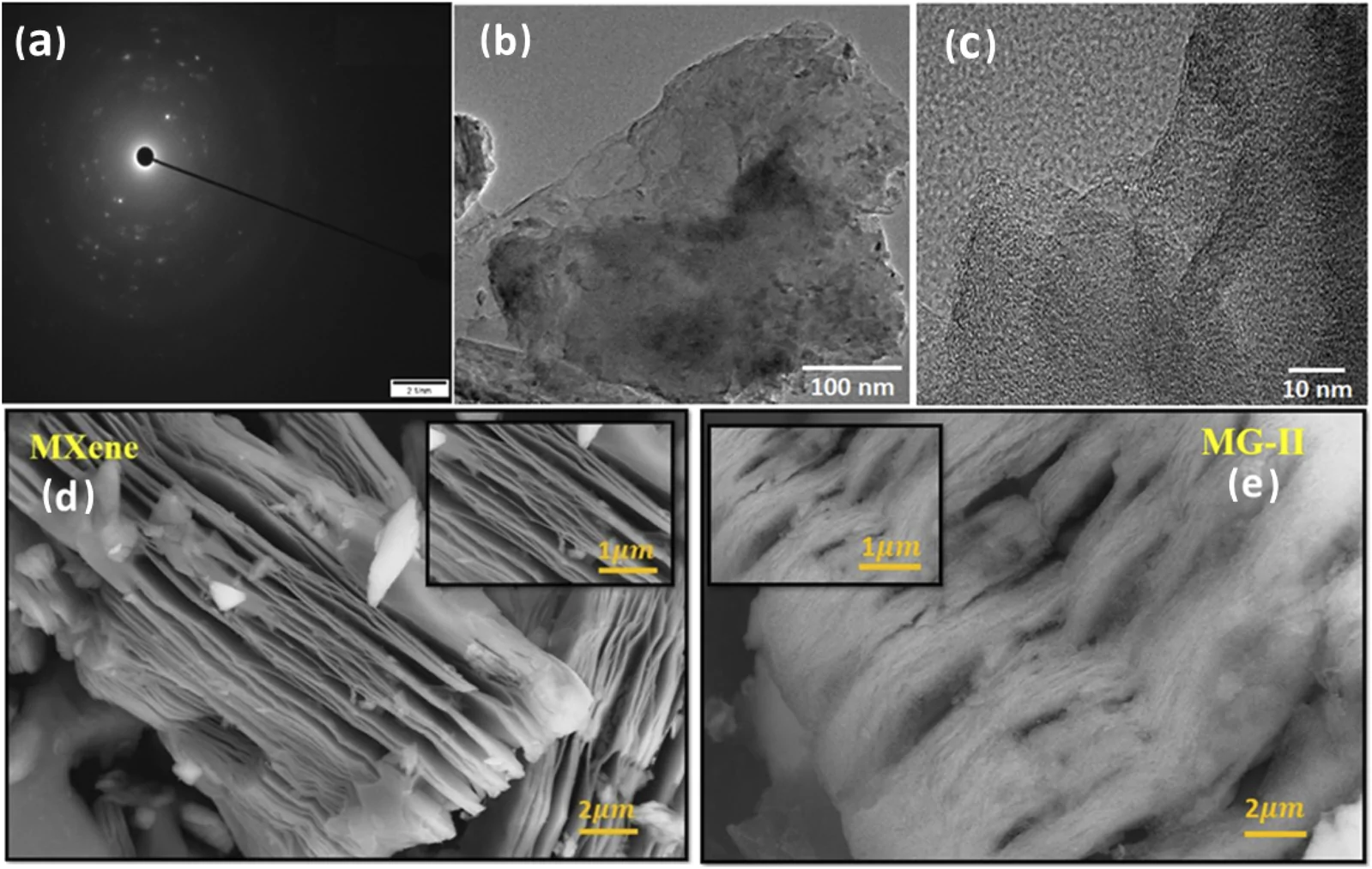
(a) Selected areas electron diffraction (SAED) and (b and c) HR-TEM image respectively of MG-II composite and (d and e) corresponding SEM images of MXene 2D sheets and MG-III composite, respectively.

This high loading of graphene results in a denser network and interlayer space, which results in improved cross-linking and strengthening effects between the two materials. Moreover, the MG-III composition is characterized by a greater dispersion of graphene flakes on the MXene layers, because the higher graphene loading partially blocks the MXene layers can be seen in Fig. S3(e). The presence of oxygen in the EDX spectra which could be attributed to the surface termination of as-synthesized MXene, graphene, and their composites. The EDX spectra of graphene, MG-I and MG-III composites are shown in Fig. S3(b, d and f). While the EDX spectra Of MXene and MG-II composite are depicted in Fig. S4(c and d), respectively. Quantitative EDX analysis (Fig. S3d, f and S4d) confirmed a low residual Al content across the samples: approximately 1.3 wt% (0.9 at%) in MG-I, 1.2 wt% (0.8 at%) in MG-II, and 1.2 wt% (0.7 at%) in MG-III, confirming substantially complete removal of Al from the Ti_3_AlC_2_ precursor regardless of graphene loading. These materials tend to absorb species from the environment where the high surface area, reactivity, and electronegativity of oxygen play a vital role. Also, the weight percentage of carbon is enhanced as the concentration of graphene increases. The XRD and SEM complemented with TEM analysis assessed composite structural stability. Its high electrochemical performance implies structural integrity.

### 3.3. Raman and FTIR analysis

Raman spectroscopy can detect molecular signatures and is responsive to minute quantities of transition metal oxides, often seen in composite structures, as well as in amorphous materials.^50^ In order to ascertain the defect density of carbon material in the composites and confirm the existence of unoxidized MXene in the composites, the material was analyzed using Raman spectroscopy in a frequency range of 100–3000 cm^−1^, as illustrated in Fig. S4(a).The peak at 174 cm^−1^ which is known as the flake region consists of in-plane (doubly degenerated E_g_) vibrations mode of Ti_2_ (surface titanium atoms) and C in the outer layers. This vibration signifies the highest quantity of atoms within the MXene unit cell. The 233 cm^−1^ peak observed in both uncoated MXene sheets and composite substances is associated with the in-plane and out-of-plane vibrations of Ti_2_ and C atoms in Ti_3_C_2_O_2_, respectively. The in-plane movements of the Ti_2_ and C atoms are assumed to be weaker by the surface functionalized sheets. Compared with a pristine MXene sheet, MXene that includes surface terminations typically has a wider *d*-spacing and a lower crystal lattice parameter “*a*”, nevertheless all atoms have the same Wyckoff locations. Furthermore, neither the MXene nor the composites' Raman spectra showed a prominent TiO_2_ peak located at 142 cm^−1^, suggesting that the MXene layers remained unoxidized to generate metal oxide nanoparticles. The disintegration and development of the segmented titanium carbide sheets corresponded with the peak expansion observed in the Raman spectra and the broadened XRD pattern.^51^

According to previous studies, the next two modes at 422 cm^−1^ and 611 cm^−1^ are related to the in-plane and out-plane (E_g_ and A1_g_) oscillation of the surface terminal (–F, –OH) groups Ti_3_C_2_(OH/F)_2_ attached due to the presence of Ti_3_C_2_ atoms, respectively.^44,50,52^ It's noteworthy that MXene oxygen-containing groups boost its capacity. In Fig. S5(a), a well-developed wide peak was also seen at 1580 cm^−1^, which is the graphitic G-band, which represents the in-plane vibration of sp^2^ hybridized carbon atoms. However, the peak's widening suggests there's a lack of numerous defects and that the carbon is amorphous (sp^3^ hybridized carbon). Moreover, it is worth noting that the all Ti_3_C_2_ MXene and graphene bands present in the MG-II composite as depicted in Fig. S4(a). This peak agrees well with earlier MXene findings from Yury Gogotsi's research team.^53^ On the other hand, the two additional peaks D- and G-spectral features at around 1347 and 1586 cm^−1^ in graphene and M/G composite materials can be seen in Fig. S5(a).^54,55^ The D-band, associated with disorder in graphene materials, is produced by the breathing modes of sp^2^ atoms located in rings. The G-band is generated by the elongation of all sp^2^ atom pairs within rings and chains. A shift in the intensity *I*_D_/*I*_G_ is worth noting when the loading quantity of graphene increases, which is in the following sequence of MG-II (0.98) > MG-III (0.95) > MG-I (0.93) > G (0.86) can be seen in Fig. S5(b). Compared to other composites, MG-II exhibits a greater disorder intensity because of the robust contact and bonding between the graphene and MXene sheets.^52,55^

The FTIR spectrum of graphene reveals multiple types of chemical bonding, such as C–O–C epoxide group bond stretching vibrations at 986 cm^−1^, aromatic C=C bending at 1737 cm^−1^, and C–O groups in carboxyl and carbonyl functionalities, along with O–H stretching vibrations at 1212 cm^−1^ and 3730 cm^−1^ (ref. 56–58) as shown in Fig. S6. Upon the extraction of the aluminum layer from the MAX phase, the MXene surface was augmented with functional groups, leading to notable peaks at 1221 cm^−1^, 1367 cm^−1^, and 1735 cm^−1^. Moreover, minor peaks at 2971 cm^−1^ and 3730 cm^−1^ are observable in Fig. S5(b). These peaks correspond to different chemical bonds, including carbon–fluorine (C–F), carbon–hydrogen (C–H), and carbon–oxygen double bonds (C=O). The peaks at 1735 and 3730 cm^−1^ are linked to molecular water, C=O, and –OH bonds, respectively.^59^ The C–H peak at 1367 cm^−1^ is greater, indicating that OH termination was introduced during the HF acid etching process.^60^ The peaks noted in the FTIR spectra of MXene, representing oxygen and fluorine-containing groups, indicate a moderate hydrophilic characteristic despite the relatively weak and less pronounced strength of the bonds involved due to their lower concentration.^49,61^ The isolated hydroxyl groups can also have stretching vibration modes that are related to Ti^4+^ cations.^62^ At 583 cm^−1^, the band relates to the out-of-plane vibrations (twisting or rocking) of Ti–C. In addition, the manifestation of an asymmetric/symmetric methyl stretching band at 2971 cm^−1^ can be linked to the existence of amorphous carbon in the samples.^49^ Similar behavior can be seen in the spectra of the composite in Fig. S6. Both graphene and MXene contains carbon. The formation of their composite would again result in the production of carbon-based aromatic or carboxylic groups. Critically, this is evident from the spectra displayed in Fig. S6, that the addition of graphene in MXene has no apparent effect on the spectra of their different concentration of composites.

### 3.4. XPS analysis

The XPS analysis offers comprehensive insights into the chemical composition of the graphene and MXene (Ti_3_C_2_T_*x*_) composites. Fig. S7 presents the XPS survey scan of the graphene and MXene composites, indicating the presence of C, Ti, O, F and Si elements only. The Si peak appeared from a substrate as the Si substrate was used for making the thin film of the sample for XPS analysis. The atomic percentage of C, O, Ti and F are 69.5%, 18.8%, 9.3%, and 2.5%, respectively. The comparatively high carbon content is consistent with the carbon-rich graphene/MXene composite, although the O and Ti signals are related primarily with the oxygen-containing and Ti-based MXene surface species. Fig. 4(a) displays deconvoluted peaks of the Ti 2p spectrum, where multiple peaks observed at 454.9 eV, 456.4 eV, 459.0 eV, 460.9 eV, 462.4 eV, and 464.8 eV, attributed to different oxidation states of titanium. The 454.9 eV and 456.4 eV peaks signify Ti–C bonds, whereas the 459.0 eV and 464.8 eV peaks are associated with spin–orbit splitting of Ti 2p_3/2_ and Ti 2p_1/2_ core levels suggesting the presence of titanium oxides. The peaks around 460.9 eV and 462.4 eV, may be allocated to C–Ti^3+^–(O/OH)/F–C–Ti.^63,64^Fig. 4(b) shows the C 1s spectrum resolved into four sub-peaks. The first peak at 284.01 eV is characteristic of C–C/C=C bonds, typical of sp^2^ hybridized carbon in graphene. C–O and C=O bonds are responsible for the peaks at 284.6 eV and 285.8 eV, indicating the functionalization of graphene with oxygen-containing groups. The peak at 288.0 eV suggests the presence of carboxyl groups (O–C=O), likely due to oxidation during the composite preparation.^65,66^ Similarly, Fig. 4(c) illustrates the O 1s deconvoluted spectrum, with peaks at 530.4 eV, 531.8 eV, and 532.9 eV. It is known that the peak of 530.4 eV corresponds to Ti–O bonds, indicative of titanium oxides. While the peaks at 531.8 eV related with C–Ti–OH_*x*_, the peak of 532.9 eV is known as the O=C–O bonds. These oxygen species highlight the modification of graphene and oxides associated with MXene surface.^67,68^Fig. 4(d) presents the F 1s spectrum, showing peaks at 685.0 eV and 686.8 eV. These peaks are indicative of C–Ti–F bonds, suggesting the presence of fluorine terminations on MXene surface. The existence of these fluorine terminations is a typical result of the synthesis process involving HF etching.^67^ The composites' graphene-MXene bonding was examined using XPS. In the Ti 2p spectra, peaks at 456.4 and 454.9 eV show Ti–C bonds, whereas peaks at 459.0 and 464.8 suggest Ti–O–C bonding. The XPS spectra shows Ti–C and oxygen-containing Ti surroundings along with C–O/C=O species, indicating chemical interface between the two components. These characteristics, combining with the DFT-predicted electronic restructuring at the Ti_3_C_2_T_*x*_/graphene interface, are aligned with interfacial linkage that can support charge redistribution.

**Fig. 4:**
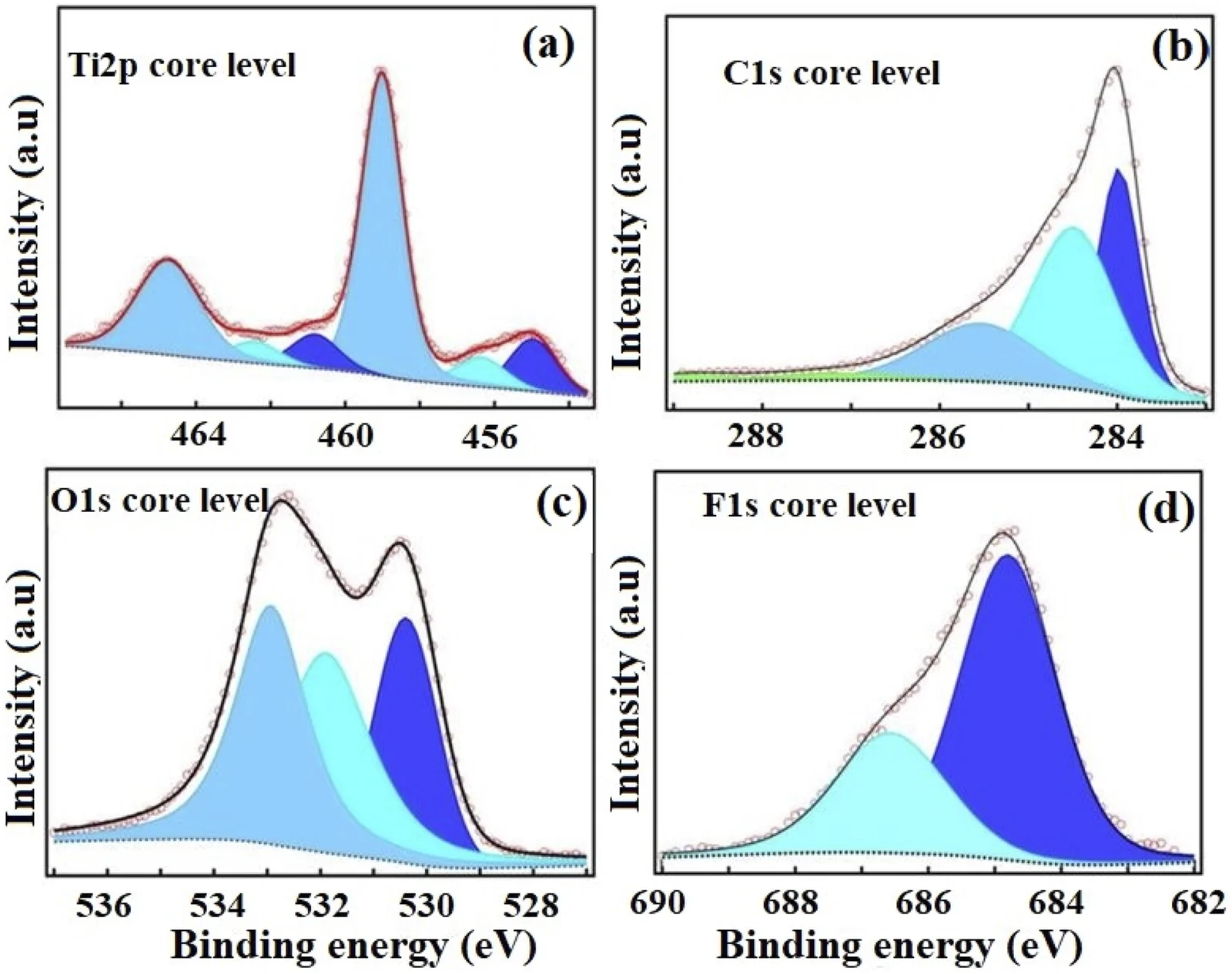
XPS high resolution scans (a) Ti 2p, (b) C 1s, (c) O 1s, and (d) F 1s for Ti_3_C_2_T_*x*_ MXene/graphene composite.

### 3.5. Electrochemical analysis

The electrochemical characteristics of the unmodified MXene, as well as the graphene, MG-I, MG-II, and MG-III, are conducted *via* multiple tests including CV, EIS, and GCD inside a three-electrode configuration of an electrochemical cell. To ensure accurate results, an electrolyte of 1M KOH solution is utilized. Fig. 5(a and c) depicts the CV graphs of the MXene and MG-II composite, whereas Fig. S8(a–c) presents the CV curves for the pristine graphene, MG-I, and MG-III samples, respectively. Fig. 5(e) illustrates the CV curves of the uncoated Ni foam and MG-II cells for 10 mV s^−1^. To investigate the role of nickel foam current collector, we performed extra CV measurements using a vacant nickel foam electrode. In comparison to the composite electrodes, the findings demonstrate that the collector's current response is negligible.^69,70^ Thus, precise electrochemical characterization is guaranteed, and the observed redox peaks are mainly attributable to the active materials, as can be seen in Fig. S9. It is seen that the Ni foam generates negligible capacitance when compared with the composite electrodes which suggest the current collector's little influence on capacitance.

**Fig. 5:**
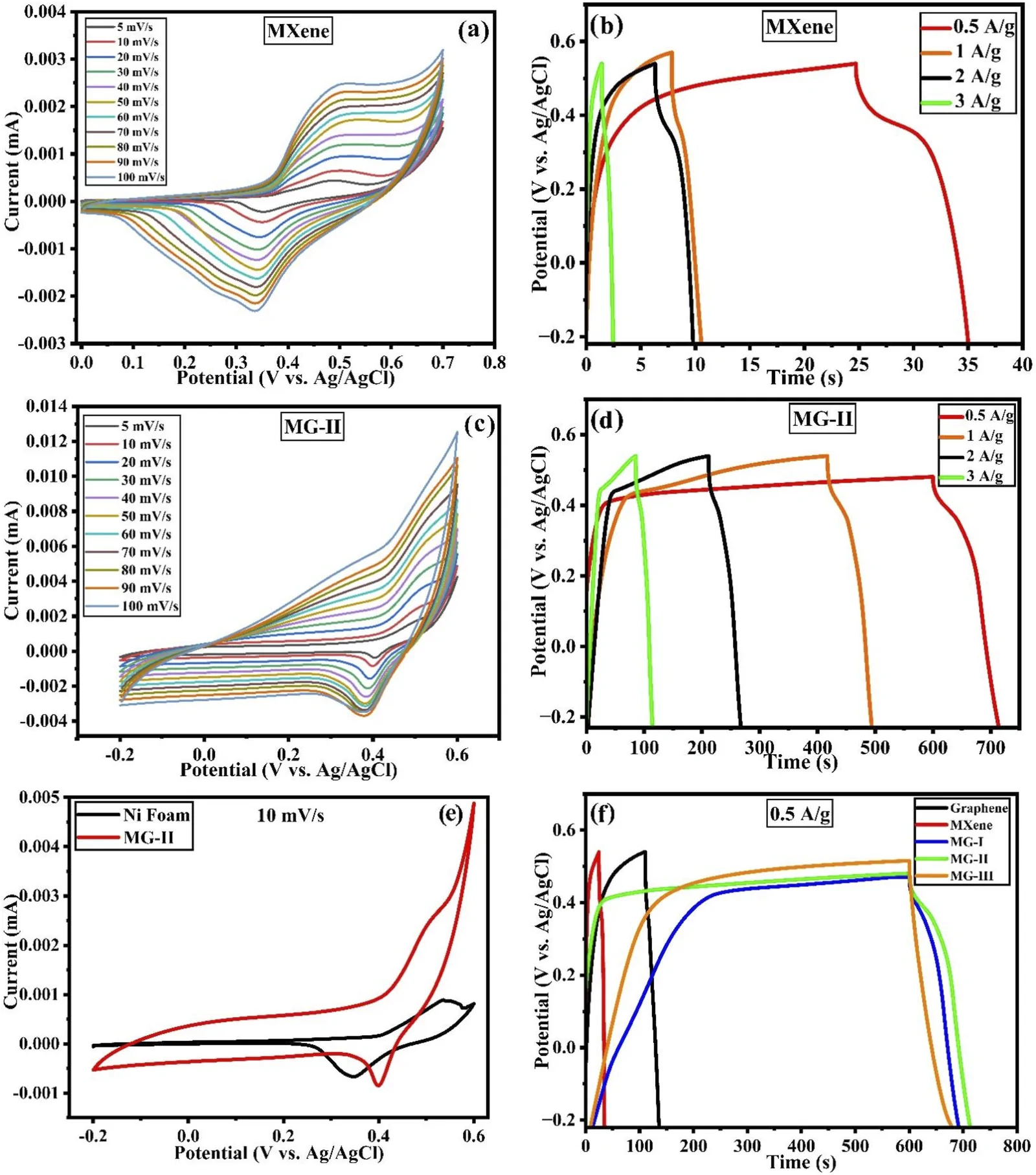
CV graphs for different scan rates and GCD plots with diverse current densities of (a and b) MXene, (c and d) MG-II composite (e) comparing CV graph of pure Ni foam and MG-II at 10 mV s^−1^ scan rate. (f) Relative GCD plot of all samples at 0.5 A g^−1^.

Interestingly, the currents from the MG-II electrode surpass those of the pure materials as well as MG-I and MG-III electrodes, signifying a notable boost in specific capacitance. The CV curves derived from the composite electrodes have notable and pronounced redox peaks, signifying the pseudocapacitive properties of the electrodes. These curves were produced utilizing various scan rates between 5 and 100 mV s^−1^ for 0–0.6 V voltage window for pure substances in Fig. 5(a) and S8(a), and −0.2 to 0.6 V for composite substances as illustrated in Fig. 5(c), S8(c and e). These redox features are consistent with the intrinsic Ti(iii)/Ti(iv) surface-oxidation-state change coupled with O/OH surface-group transformation established for Ti_3_C_2_T_*x*_ by first-principles studies.^71^

Similar to Fig. 5(a), the CV curve MG-II displays a greater area than that of both composite MG-I and MG-III because of the increasing amount of graphene, indicating improved capacitive behavior and high-rate charge storage. Because Ti_3_C_2_T_*x*_ displayed higher electrochemical activity relative to graphene owning to its favourable attributes of high metal-like conductivity and hydrophilic nature. All curves have distinct redox peaks, which clearly illustrate pseudocapacitive behaviors. Moreover, the CV curves maintain their form consistently even when subjected to rapid scan rates, demonstrating exceptional high-rate efficiency.^43^ The primary reason behind the slight fluctuation observed in the redox peaks between the composite and pristine materials could be attributed to the structural disparity that exists between the samples. The individual capacitance of each electrode, as inferred from the CV curves, was assessed using the eqn (4) and is displayed in Table 1.4
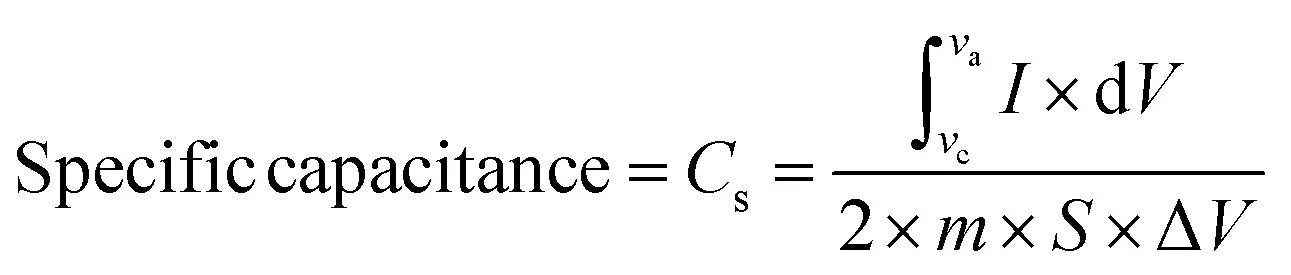


**Table 1:** The specific capacitance of pristine and composite materials evaluated using GCD profiles at diverse current densities

Materials	0.5 A g^−1^	1 A g^−1^	2 A g^−1^	3 A g^−1^
Graphene	124.8	51.3	22.2	26.7
MXene	138.8	46.1	18	9.2
MG-I%	328.9	235.3	122.7	101.1
**MG-II%**	**366.3**	**298.9**	**264.1**	**238.4**
MG-III%	247.5	154.3	103.8	99.08

Conversely, the specific capacitance of both pure and composite materials was assessed *via* GCD testing across a voltage span of −0.2 to 0.6 V at varying particular current densities of 0.5, 1, 2, and 3 A g^−1^, as depicted in Fig. 5(b, d), S8(b, d and f).

It is clear that when current density increases, the material's discharge period is much shorter. The redox reaction's sluggish kinetics may explain this phenomenon compared to the potential's quick changes.^72^ Many variables, including electronic conductivity, pore size, and distinct surface region, can affect an electrode material's specific capacitance.^73^ It is noted that the discharge time was significantly longer in the composite MG-I, MG-II and MG-III electrodes as compared to pure graphene and MXene electrode materials. This indicates the permeable structure was formed avoided and hindered the stacking between the graphene and Ti_3_C_2_T_*x*_ MXene sheets including the self-assembly of Ti_3_C_2_T_*x*_ nanosheets, which assisted to some extent to improve capacitance and promote electron diffusion and ions transport. According to the eq. (*C*_sp_ = *I*Δ*t*/*m*Δ*V*), the specific capacitance (*C*_sp_) of composite materials named MG-I, MG-II, and MG-III was calculated. This can be seen that the MG-II composite has greater capacitance 366.3 F g^−1^ than other composite MG-I (328.9 F g^−1^), MG-III (247.5 F g^−1^) and the bare materials graphene (124.8 F g^−1^) and MXene (138.8 F g^−1^) at rate of 0.5 A g^−1^ as displayed in Fig. 5(f), significantly exceeds the findings of the prior study.^74^ Conversely, the GCD patterns at varying current densities span from 1 to 3 A g^−1^, as illustrated in Fig. 5(d), MG-II performed well at 3 A g^−1^. The composite's robust charge/discharge performance and structural integrity at high current density were shown after 5000 cycles when it retained 85.39% of its capacitance.

In a comparable investigation, the MXene/graphene composites detailed by Shao *et al.*^75^ demonstrated remarkable electrochemical energy storage capabilities of 233 F g^−1^ at 1 A g^−1^; however, the present study reveals that the Ti_3_C_2_T_*x*_ graphene composite attains a notably high capacitance of 298 F g^−1^ at 1 A g^−1^ with high cycling stability. Comparing the performance of various akin family of composite materials, our study highlights the best composite material combination for supercapacitor applications. The main causes of the voltage differences seen in the GCD profiles across diverse current densities are the electrochemical polarization effects,^76^ the charge transfer kinetics, and the internal resistance (IR) decrease. The higher current flowing through the electrode causes a more noticeable *IR* drop at higher current densities which in turn causes a lower plateau voltage for discharge. Based on the GCD profiles, the *IR* drop was approximately 45, 90 and 108 mV at 0.5, 1 and 2 A g^−1^, respectively, corresponding to only 5.6 to 13.5% of the operating voltage window. This gradual increase in *IR* drop with increasing current density is expected and is consistent with the ohmic relationship *IR* = *I* × ESR, where a higher applied current amplifies the voltage loss across the equivalent series resistance of the electrode/electrolyte interface, as widely reported for supercapacitor electrodes.^77^ The charge–discharge cycles for high current application can also lower capacitance by limitation of ion transport in the electrode which leads to inadequate utilization of active sites.^78^ These combined effects account for the discrepancies in the GCD curves, and comparable patterns have been documented in earlier research on supercapacitors based on MXene.^79^ In addition, the specific capacitance for the pure and composite electrodes, as determined from CV and GCD analyzes at varying scan velocities and current densities, is presented in Fig. 6(a and b) and summarized in Tables 1 and S2, separately.

**Fig. 6:**
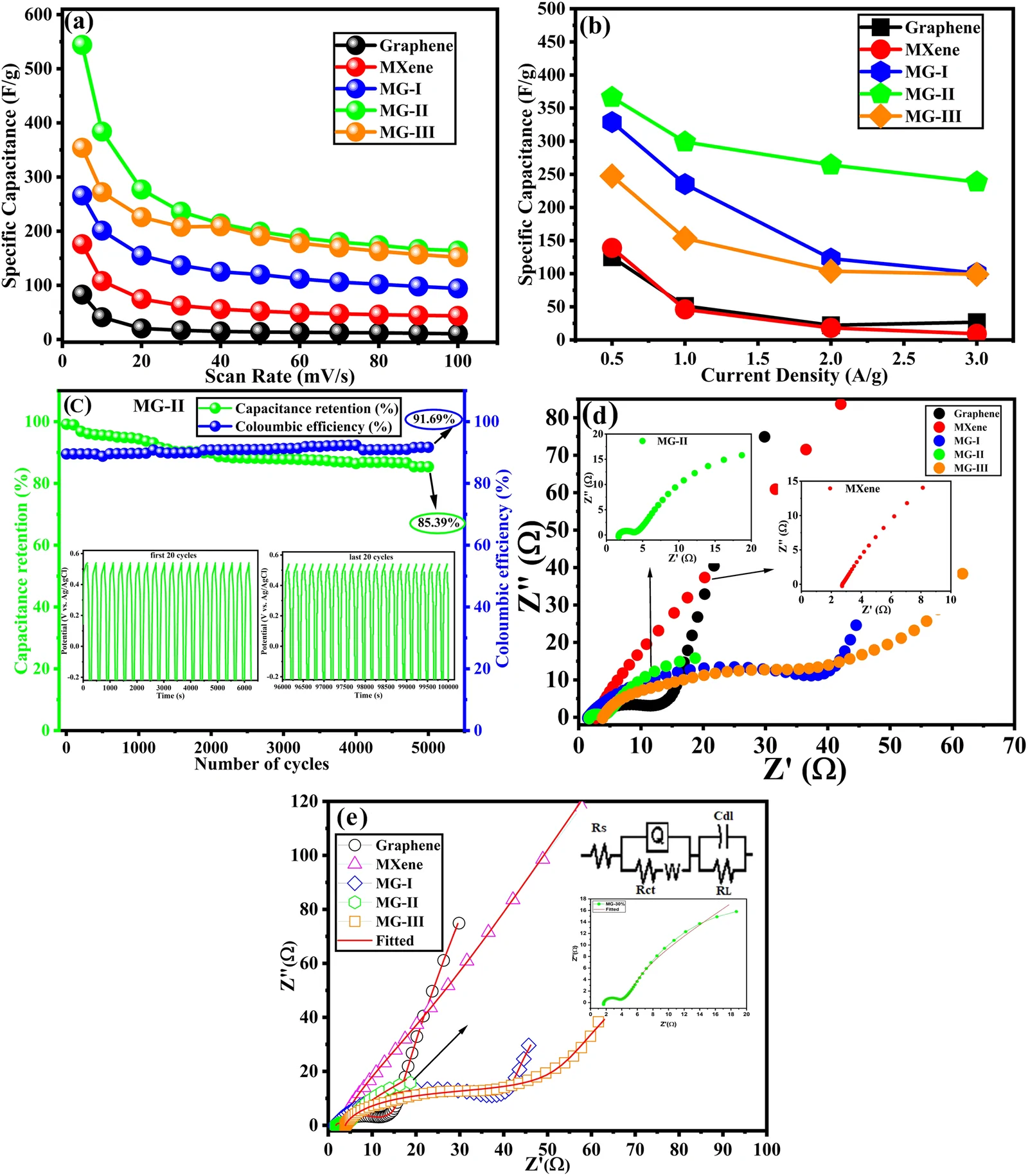
(a) A graph depicting specific capacitance in relation to different scan speeds, (b) graph of specific capacitance in relation to current density, (c) a graph illustrating the correlation between the number of cycles and capacitance retention as well as coulombic efficiency for MG-II at a current density of 1 A g^−1^, (the inset graph shows the first and last 20th cycles of the charge–discharge). (d) Nyquist plot; the inset displayed the plot of MXene and MG-II composite, (e) Nyquist and fitted plot; the inset proposed the equivalent circuit.

This illustrated that the electrodes' capacitance lessened as the scan rates and current density escalated, because of its characteristics, which include resistance within, polarization, and the insertion and elimination of electrolytic ions owing to mechanical stress. Additionally, a three-dimensional graph of particular capacitance, calculated from CV and GCD, at various scan speeds and current densities, can be seen in Fig. S10(a and b). In both situations, the specific capacitance of composite electrodes is higher than that of the pure graphene and MXene electrodes, respectively. Furthermore, the GCD profile of bare nickel foam (Fig. S15) reveals essentially negligible capacitance, indicating that the MXene/graphene combination is responsible for the electrochemical performance.

#### 3.5.1. Trasatti method

In addition, CV investigation revealed that the redox process is governed by diffusion, a characteristic that could be crucial for the pseudocapacitive behavior noted at elevated scan rates. The Trasatti and Dunn differentiation methods were used to investigation of the electrode mechanism such as diffusion-controlled and surface-controlled formation. The value of *b* in power law *i*_p_ = *av*^*b*^ distinguish between diffusion and surface control process. When *b* equals 0.5, a diffusion-governed faradaic mechanism transpires; conversely, when *b* equals 1, surface redox reactions lead to a non-diffusion-governed process. The Trasatti approach is the most straightforward of the two examined techniques for explaining the electrode's mechanism.^80^

The surface- and diffusion-controlled charge-storage characteristics were analyzed *via* Trasatti method in which the total charge capacity (*C*_T_) is comprised of the inner surface charge (faradaic process) and the outer surface charge (non-faradic process) (*C*_T_ = *C*_i_ + *C*_o_). It just permits the surface mechanism to occur since, generally, *V* → ∞ in the outer surface. The site of interaction between the electrode and the electrolyte is therefore the more convenient site. Likewise, at the internal surface *V* → 0, allowing ample time for diffusion and further ionization of ions, reducing the available surface area. The maximum total capacitance (*C*_T_) may be calculated from the linear correlation between (*v*) 1/2 and (1/*C*_sp_) in the Cottrell eq. if the assumption of a semi-infinite linear diffusion of ions is used and the *y*-intercept of the eq. is calculated as shown below eqn (5).5
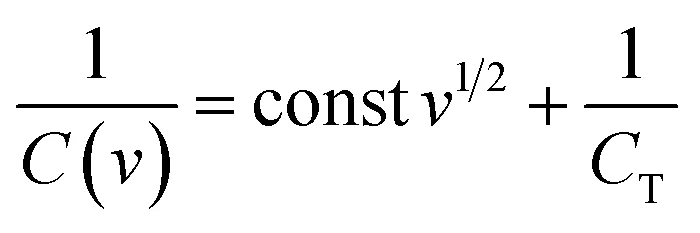

6 *C*(*v*) = const(*v*)^−1/2^ + *C*_o_

A linear relationship was observed between (*v*) 1/2 and the inverse of the specific capacitance of the electrodes being studied, as displayed in Fig. S11(a–e). The intercepts on the plot correspond to the total capacitance, denoted as *C*_T_. Similarly, if one assumes linear ion diffusion in the semi-infinite linear mode according to the Cottrell eq. then a linear approximation plot of (*C*_sp_) *vs.* (*v*) −1/2 may be employed to determine the charge on the exterior surface (*C*_o_).

Fig. S11(f–j), shows how the square root of the scan speed (*Ψ*) correlates with its inverse are linearly fitted to the specific capacitance. The CEDLC has been identified based on the graph intercepts. Applying the formula *C*_i_ = *C*_T_ − CEDLC, the pseudocapacitive behavior is calculated. The detected non-linearity in the Trasatti plots may result from departures from the ideal semi-infinite diffusion hypothesis, linked to diverse pore architectures, limited ion diffusion, and pseudocapacitive charge-storage mechanisms.^81,82^ The neat sample graphene showed 20% and 80% capacity corresponding to the contributions of the surface and diffusion mechanism, pure MXene exhibited 35% and 65% capacity according to the contributions of the EDLC and PC, and MG-II provided 40% and 60% capacity based to the contributions of the EDLC and PC. Additionally, a graphical comparison of the individual capacitances of every electrode under investigation at different scan speeds is shown in Fig. S11(k).

The percentage of capacitive contribution was estimated through the eqn (7) and (8).7
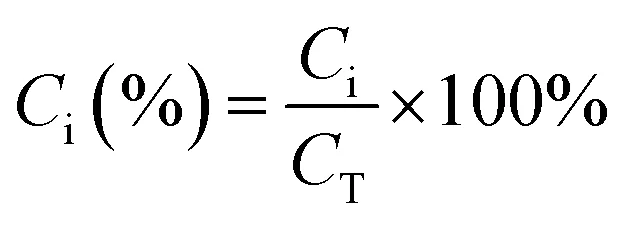
8
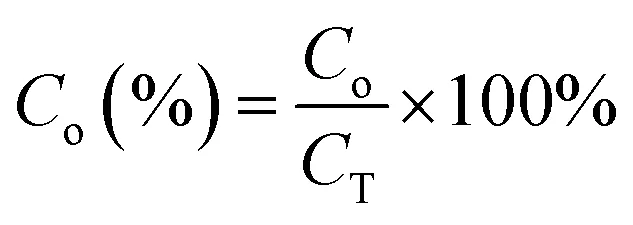


#### 3.5.2. Cycling stability

In Fig. 6(c), S12(a and b), the MG-I, MG-II, and MG-III electrodes showed outstanding long-term cycle durability, maintaining a specific capacitance after 5000 charge–discharge cycles at a current density of 1 A g^−1^, indicating minimal structural degradation. The capacitance retention of the MG-I, MG-II and MG-III electrodes was achieved at 80.31%, 85.39% and 78.03% are represented in Fig. 6(c), S12 (a and b). Additionally, the MG-I, MG-II and MG-III electrode materials exhibited a notable coulombic efficiency ratio of 88.56%, 91.69% and 86.69%, with no significant difference observed between the first 20th cycles and the last 20th cycles, underscoring its impressive electrode stability. The coulombic efficiency and capacitance retention of the MG-II electrode exhibited outstanding electrochemical performance in comparison to alternative electrode materials, and was assessed over 5000th cycles as tabulated in Table 2. The 30 wt% composition (MG-II) was therefore defined as optimal based on its combined performance across all measured parameters, including the highest specific capacitance (366.3 F g^−1^ at 0.5 A g^−1^, *versus* 328.9 and 247.5 F g^−1^ for MG-I and MG-III), the lowest charge-transfer resistance (*R*_ct_ = 2.79 Ω), and the highest capacitance retention (85.39% after 5000 cycles, *versus* 80.31% and 78.03% for MG-I and MG-III), rather than any single parameter alone. The calculation of coulombic efficiency was carried out utilizing a specific eq.;9
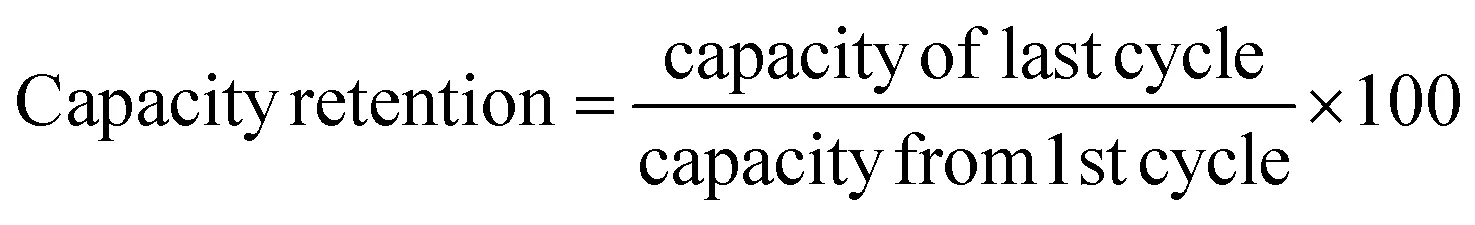
10
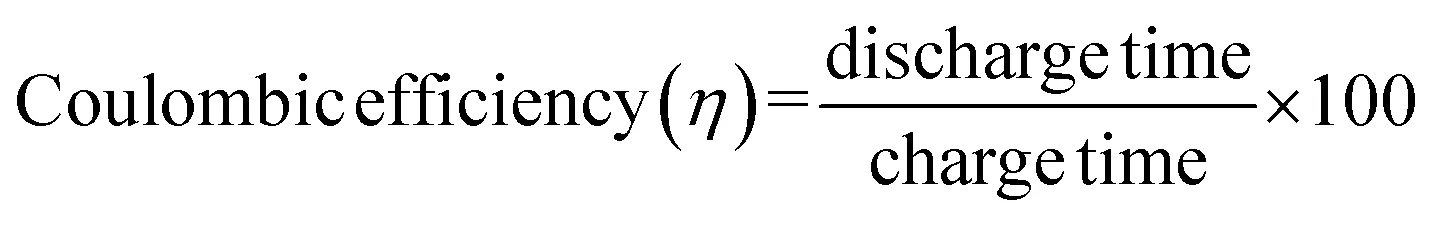


**Table 2:** The electrochemical performance of constructed MXene/graphene electrodes is compared with results from various literature publications

Materials	Synthesis methods	Specific capacitance (F g^−1^)	Energy density (Wh kg^−1^)	Power density (W kg^−1^)	Capacitance retention (%)	Electrolyte	Ref.
MXene/rGO aerogel	Simple ultrasonication method	233 F g^−1^ at 1 A g^−1^	N/A	N/A	91.01% after 10 000 cycles	1 M H_2_SO_4_	75
MoS_2_/graphene	One-step electrochemical exfoliation	227 at 0.1 A g^−1^	N/A	N/A	89% after 2000 cycles	1 M Na_2_SO_4_	43
d-Ti_3_C_2_/rGO	Hydrothermal method	247 F g^−1^ at 1 A g^−1^	15.7	3738.7	90% after 5000 cycles	PVA/H_2_SO_4_ gel	83
Ti_3_C_2_T_*x*_@CNTs	*In situ* precipitation method	201 F g^−1^	14	486	78.1% after 10 000 cycles	6 M KOH	84
C@Ti_3_C_2_	*In situ* condensation polymerization process	250.6 F g^−1^ at 1 A g^−1^	10.8	600	94% after 5000 cycles	6 M KOH	85
MXene/CNTs film	Vacuum filtration process	300 F g^−1^ at 1 A g^−1^	N/A	N/A	92% after 10 000 cycles	1 M H_2_SO_4_	86
Ti_3_C_2_T_*x*_/RGO-7	Hydrothermal method	154 F g^−1^ at 2 A g^−1^	N/A	N/A	85% after 6000 cycles	2 M KOH	87
**Ti**_**3**_ **C**_**2**_ **T**_***x***_ **/graphene**	***In situ* precipitation method**	**75.5 F g^−^** ^**1**^ **at 1 A g^−^** ^**1**^	**41.95**	**1000.1**	**86.6% after 10 000 cycles**	**1 M KOH**	**This work**

#### 3.5.3. Electrochemical impedance spectroscopy

For a better understanding of the electrode–electrolyte interfacial and their capacitive behaviors, electrodes underwent EIS testing, which was conducted in the frequency range of 0.1 Hz to 100 kHz at an open circuit potential (OCP) and an excitation signal amplitude of 5 mV for a detailed study of the physical parameters. In Fig. 6(d and e), the data has been plotted between the real and imaginary impedance, which displayed two separate distinct Nyquist plots; one is a low-frequency linear segment and the other is a high-frequency semicircle. The Nyquist plots acquired display a semi-circle at higher frequencies, indicating the existence of electronic resistance within the electrode materials. Table 3 contains the circuit model's fitting parameters that were employed. This equivalent circuit model comprises a solution resistance (*R*_S_), charge transfer resistance (*R*_CT_), Warburg impedance (*W*), double layer capacitor (*C*_dl_), and constant phase element (CPE). Furthermore, the slope of the inclined line in the low-frequency portion serves as an indicator of the electrolyte diffusion within the electrode materials.^87^ The overall impedance of the supercapacitor comprises electrical and ionic components. In addition to the interfacial resistances that exist between the particles and the current collector, the intrinsic electronic resistance of the particles is also responsible for the electronic contribution. In contrast, the ionic contribution relates to the resistances of the electrolyte in pore walls and the diffusion resistance that ions suffer when they permeate tiny pores. The equivalent circuit fitted model, *R*(*Q*(*RW*))(*CR*) can be easily seen in Fig. 6(f). In the comparable circuit model, the solution resistance *R*_s_ is arranged in series with additional components as current traverses the electrolyte across all frequencies. Constant phase elements (CPE, *Q*) are utilized to represent non-ideal capacitors caused by interface inhomogeneity or roughness. At high frequencies, current flows through *Q* following *R*_s_, while at lower frequencies, current passes through *R*_CT_ as *Q* impedance increases, forming a parallel arrangement. In addition, the impedance curve (*W*) of MG-II shows a steeper slope compared to graphene, indicating faster ion transport and less diffusive resistance, and then placed in series with another parallel arrangement of *C*_dl_ and *R*_L_ (leakage resistance) which is not important and ignored, representing a pseudocapacitive setup within the electroactive material.^88^ Moreover, the Fig. S13(c) displayed the Bode plot illustrating the relationship between the phase angle and the logarithm of frequency response inside the system. It is clear that MG-II has phase angle of at −39.5° closely resembles the phase angle of (−45°) indicating a consequence of a diffusion-constrained process.

**Table 3:** Physical parameters of materials calculated from the EIS data

Materials	*R*_s_ (Ω)	*R*_ct_ (Ω)	*Y*_o_ S s^*n*^	*n*	*W* S s^5^	*C*_dl_ (mF)	*R*_L_ (Ω)
Graphene	3.44	8.1	0.01861	0.373	11.9	26.98	0.5483
MG-I	1.44	22.6	0.02762	0.413	38.4	234	29.6
MG-II	1.18	2.79	0.06274	0.369	3.9	844.6	0.0018
MG-III	3.83	25.7	1.2 × 10^−8^	0.158	40.1	177	18.5
MXene	2.78	3.14	0.00034	0.840	0.3 × 10^−3^	120.5	6.657

It is also worth noting that the MG-II composite electrode demonstrates smaller values for *R*_s_ (1.18 Ω), addressing the high conductive properties of the MXene and *R*_CT_ (2.79 Ω) is lower in comparison to other electrode materials. The presence of these low resistance values is advantageous, as they facilitate rapid charge transfer and expedited ion diffusion, ultimately enhancing the electrochemical performance. A detailed analysis of complex capacitance was conducted to gain more understanding and also to demonstrate how the specific capacitance changes with frequency can be seen in Fig. S13(d–f). The below eqn (10) defines capacitance, which is recognized in EIS, featuring both a real and an imaginary component.

11 *C*(*ω*) = *C*′(*ω*) + *C*″(*ω*)

The symbols *C*′ and *C*″ signify the complex capacitance of the actual and imaginary components, respectively, with *ω* equating to 2π*f*. The following eqn (11) and (12) are used to specify these components further.12
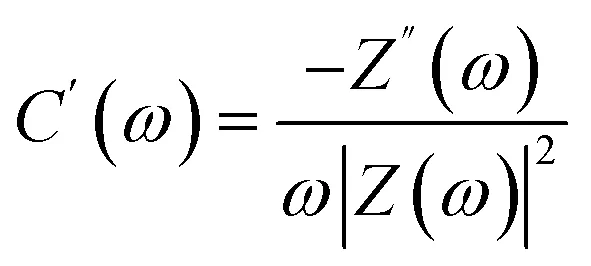
And13
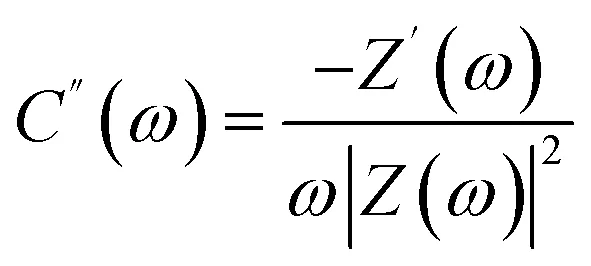


The real part offers capacitance or charge retention, but the imaginary part leads to energy loss or inefficiencies in the electrochemical mechanism of several processes like ion or charge movement, among others. Ideal capacitance has an imaginary element that is either zero or missing, indicating good charge storage, and a real part that stays constant. Nonetheless, in an actual system (Fig. S13(d)), the capacitance remains constant (at low frequencies) before beginning to deviate from a linear trajectory as the frequency increases.

The cause of this divergence may be variously identified as resistance to ion diffusion, resistance to charge transfer, or electrochemical processes occurring at the electrode surface. The frequency-dependent change in the real capacitance component indicates the degree to which electrolyte ions enter the porous structure of materials. At reduced frequency ranges, ions can penetrate the pores of the electroactive material, leading to an elevated *C*′(*ω*) due to enhanced accessibility. However, at higher frequencies, ions are constrained to only accessing the electrode's surface, leading to a decrease in *C*′(*ω*). The EC system exhibits resistive behavior at very high frequencies, and *C*′(*ω*) becomes a frequency variable. As a result, it is clear that charge accumulated in the system is delivered *via C*′(*ω*) at lower frequencies. The imaginary aspect *C*(*ω*) of capacitance changes with frequency, revealing that *C*(*ω*) reaches its maximum value at a certain frequency (*ψ*).

The reciprocal of (*ψ*) yields *τ*_o_, the relaxation time parameter of the system's dielectric materials.^89,90^ In the current investigation, this period is revealed to be 0.99 s, demonstrating the rapid charge–discharge characteristic response and strong electrochemical capacitance characteristics, *τ*_o_ is occasionally termed a supercapacitor performance metric.^91^ The EIS findings indicate that the electrochemical performance of the MG-II electrode surpasses that of other compositions owing to attributes like phase purity, diminished agglomeration, enhanced EDLC and PC contributions, lower solution resistance *R*_s_, improved electrolyte ion infiltration, and decreased dielectric relaxation time constant. All of these factors indicate MG-II electrode has good electrochemical behavior when compared to all other compositions. The following formula as mentioned in eqn (14) was used to get the relaxation time (*τ*_o_) from the frequency (*f*) associated to the peak of the imaginary part (−*Z*″) in the Nyquist plot.^92,93^14
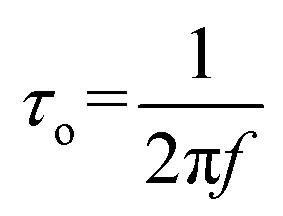


## 4. DFT calculations

### 4.1. Structural properties

In this study, we have experimentally validated different compositions Ti_3_C_2_–graphene hybrids. The bulk Ti_3_C_2_ possesses a hexagonal unit cell characterized by the space group *P*6_3/*mmc*. Fig. 7(a) illustrates the optimal bulk Ti_3_C_2_ configuration with lattice parameters: *a* = *b* = 3.06 Å and *c* = 15.11 Å, along with *α* = *β* = 90° and *γ* = 120°, which is consistent with the reported/calculated lattice parameters. The hexagonal lattice constant of graphene is *a* = *b* = 2.478 Å, exhibiting a lattice discrepancy of around 19% following the formation of a hybrid structure.

**Fig. 7:**
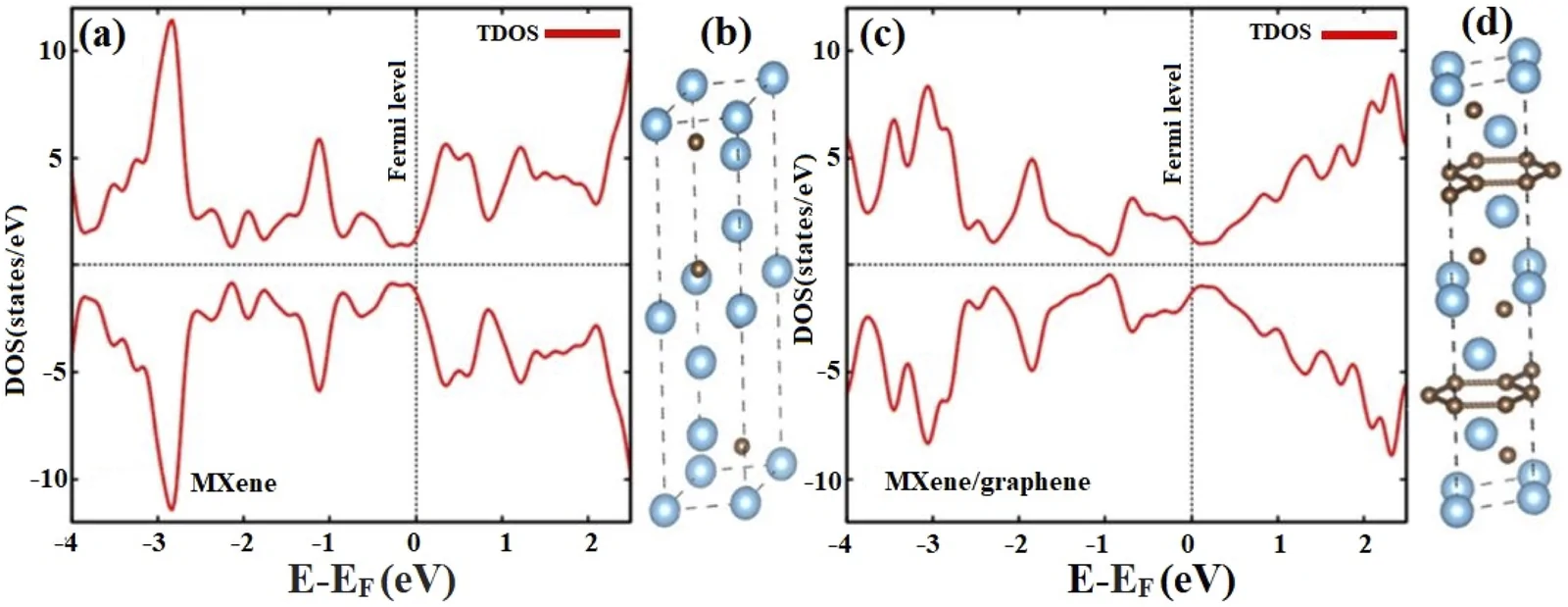
(a) Total density of states (TDOS) and optimized crystal structure (100) view of (a and b) bulk Ti_3_C_2_ and (c and d) Ti_3_C_2_–graphene hybrid composite.

This study models the properties of 15% and 30% graphene forming a Ti_3_C_2_ hybrids using supercells to represent the material structure using the first-principles calculations, we simulate 45% graphene and form a hybrid Ti_3_C_2_ by adding 2 layers of graphene within the Ti_3_C_2_T_*x*_ structure as presented in Fig. 7(d), which shows the lowest energy configurations.

### 4.2. Electronic properties

To comprehend the relationship between Ti_3_C_2_ and graphene, we have illustrated the total density of states (TDOS) for both bulk Ti_3_C_2_ and the Ti_3_C_2_–graphene hybrid, as depicted in Fig. 7(a) and (c). The DFT calculations were employed on an idealized Ti_3_C_2_ slab/bulk model without considering –O/–OH/–F terminations.

Bulk Ti_3_C_2_ shows metallic behavior. With the addition of graphene, an increase in the DOS at the Fermi level can be seen in Fig. 7(c) and the justification behind the augmentation in DOS is observable in Fig. S14. We further analyze the decomposed orbital contribution for Ti-3d of Ti_3_C_2_ and C-2p graphene for both undoped and graphene-doped MXene film in Fig. 8(a–d), respectively. The Ti-3d orbital of bulk MXene has a greater number of accessible states close to the Fermi level, which diminishes considerably in the graphene-doped configuration, indicating charge transfer from the Ti-3d orbital. On the contrary, we observe an increase in the graphene 2p orbitals in the hybrid structure, indicating charge accumulation on graphene. The transfer of charge from Ti to graphene in a hybrid is evident in the charge density difference plot illustrated in Fig. 8(e and f), corroborating the PDOS findings.

**Fig. 8:**
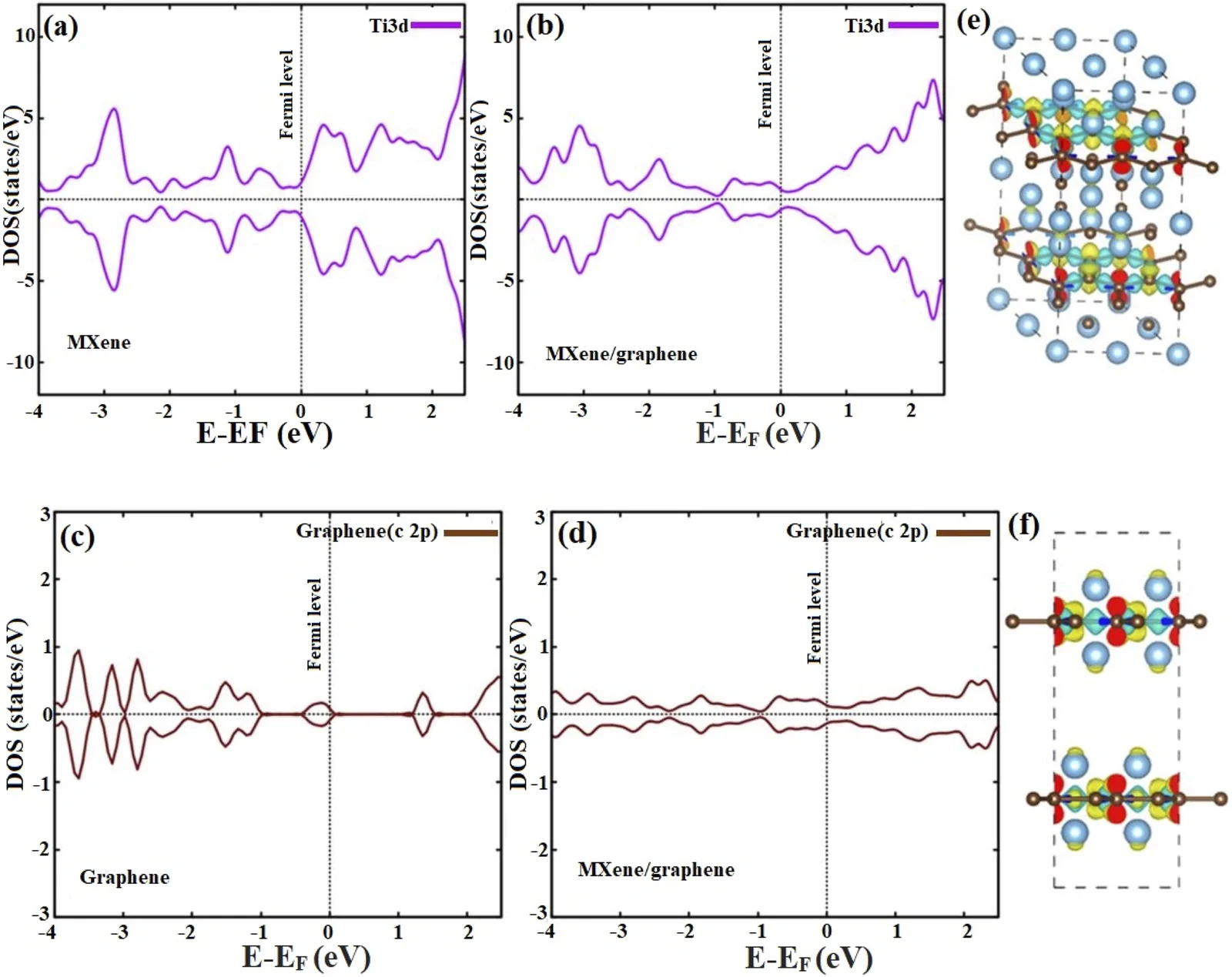
Partial density of states of 3d orbital of titanium in (a) bulk Ti_3_C_2_ (b) Ti_3_C_2_–graphene composite. Partial density of states of graphene (C 2p) of (c) bulk graphene and (d) Ti_3_C_2_–graphene composite. (e and f) Charge accumulation and charge depletion are visible through the charge density difference plot of Ti_3_C_2_–graphene hybrid.

## 5. Asymmetric device performance

Keeping in view the best performance of MG-II composites among others, the asymmetric supercapacitor was fabricated with AC as anode and MG-II composite as cathode with 1M KOH and electrochemical performance were evaluated to examine its practical prospects for energy storage uses. The fabricated MG-II device is displayed in Fig. 9(a). The CV measurement results of AC (−1.0 ∼ 0 V) and MG-II (−0.2–0.6 V) employing sweep rate of 10 mV s^−1^ displayed in Fig. 9(b). Selecting a suitable operating voltage window (*V*) for supercapacitor device has major influence on overall device's electrochemical efficiency and its use in real world applications. Also, it should be noted that electrode materials for asymmetric supercapacitors must operate within an extensive possible range in the identical electrolyte this is due to the fact that as the cell's voltage range increases, the energy density of the assembled device.^94^ So, the MG-II//AC-ASC supercapacitor's working potential window was optimized by recording the CV and GCD curves within a prospective range of 0.8 to 2 volts. Fig. 9(c and d) correspondingly display the pertinent CV plots at 20 mV s^−1^ and GCD plots at 1 A g^−1^. The CV and GCD curves exhibit no discernible changes with the expansion of the potential window; thus, a range of 0–2 V has been chosen for subsequent examination. The CV curves with increasing scan rates are illustrated in Fig. 9(e). The CV curves have shown prominent redox peaks, are indicative of a pseudocapacitive charge storage process.^95^ The uniformity of the CV curves at various scan rates implies that the MG-II//AC-ASC demonstrates commendable reversibility.^96^

**Fig. 9:**
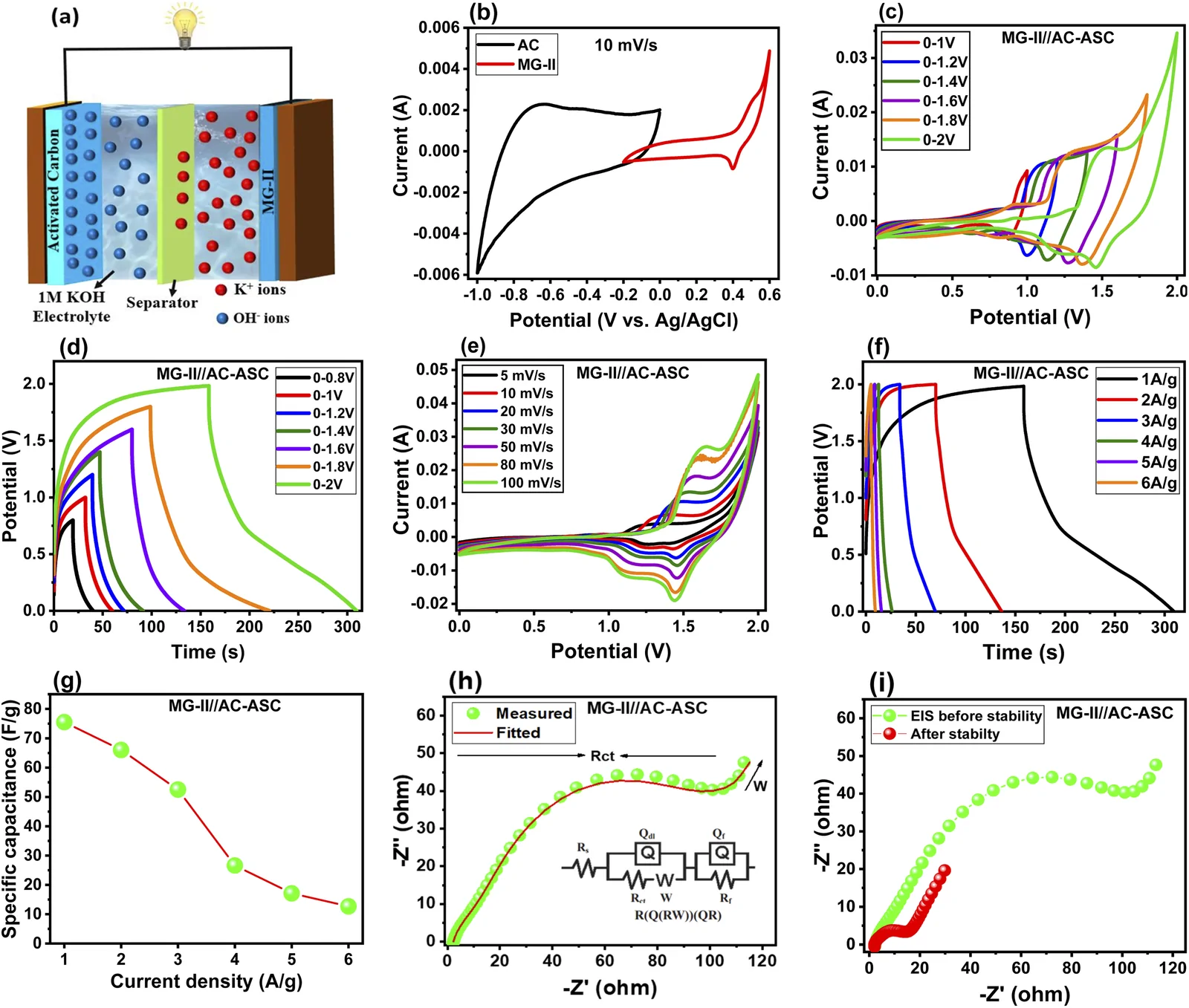
(a) Fabricated MG-II//AC-ASC device, (b) CV profile for MG-II and AC electrode at 10 mV s^−1^, (c and d) CV and GCD results of assembled MG-II//AC-ASC device with varying potential (0 to 2 V), respectively, (e) the obtained CV with varying sweep rates (10–100 mV s^−1^) at 0.2 V, (f) GCD plots at different current densities, (g) the corresponding specific capacitance *vs.* current density, (h) EIS Nyquist spectra along with the fitted circuit, and (i) EIS plots before and after the cyclic stability.

The GCD plots across diverse current densities are shown in Fig. 9(f). As the GCD is more sensitive to potential window, there is some non-linearity and asymmetry in the GCD plots illustrated in Fig. 9(d) and (f), presenting the pseudocapacitive nature. This nonlinearity or small deviation from the ideal triangular behavior is mostly attributed to the *IR* drop during discharge process. This effect of non-linearity becomes more prominent with the rise of current densities and is generally unavoidable because of internal resistance of electrode materials and electrode-current collector interface commonly occur in aqueous electrolytes as previously reported in similar MXene based composite materials.^97^ Also, there is inverse relationship between potential and specific capacitance. Fig. 9(g) illustrates the relationship between the computed specific capacitance and the current density. Notably, at 1 A g^−1^, the MG-II//AC device realized its highest specific capacitances of 75.5 F g^−1^. More significantly, even under greater current density of 6 A g^−1^, the capacitance of 12.67 F g^−1^ was preserved, indicating the good rate capacity of the MG-II//AC-ASC device. The combined effect of the EDLC type AC electrode and the redox type MG-II electrode should have produced the desired electrochemical characteristics. A lower specific capacitance value results from increasing current density attributable to the limited faradaic redox reaction occurring at the electrode's surface. A larger capacitance value is produced at lower current densities because there is a greater chance of ion adsorption and intercalation. Elevating the current density value confines electrolyte ions to the surface of the electrode, which reduces the chances of adsorption or material penetration.^98^ However, GCD offers a realistic evaluation that considers the impact of the voltage reductions and aids in forecasting the material's practicality. To govern the charge kinetic properties, the AC impedance studies of MG-II/AC-ASC were also conducted. In Fig. 9(h) the Nyquist plot, before taking the CV and GCD analysis shows that the MG-II//AC-ASC electrode material display a clear semicircle in elevated-frequency zone, which is due to their high internal charge transfer resistance (*R*_ct_). The Warburg impedance, also known as the diffusion resistance, is shown as a straight line that begins above 45° in the low-frequency domain. The Fig. 9(h) also contains the fitted Randle circuit *R*(*Q*(*RW*))(*QR*) by Zsimp Win software. Here in, *R*_s_ represents the resistance of the electrolyte, *R*_ct_ and *R*_f_ denote charge transfer resistance associated with adsorption and intercalation processes into the surface of materials, respectively and *W* is Warburg diffusion impedance. An electrical capacitor is the double layer that exists between the electrolyte and the charges. *Q*_dl_ and *Q*_f_ are identified as constant phase elements (CPE), referring to modified double-layer capacitance and material capacitance, respectively.^99^

The EIS comparison of the MG-II/AC-ASC device prior to and after cycling is displayed in Fig. 9(i). It shows that after cycling, the impedance mode is significantly decreased, and the *R*_s_ value decreases from 2.14 Ω to 1.97 Ω, while the *R*_ct_ value diminishes from 97.81 Ω to 12.65 Ω. The dispersal of material flakes and the rise in porosity are probably responsible for the decrease in electrochemical impedance of MG-II//AC-ASC.^100^

The charge storage behavior of MG//AC-ASC supercapacitors were thoroughly investigated *via* the power law eq. by scrutinizing CV curves at different scanning speeds and positing a direct association between current (*I*) and scan rate (*v*), represented as *I* = *av*^*b*^.

15 log(*i*) = log(*a*) + *b* log(*v*)

In this context, *a* and *b* represent two variables that may be changed; the values of *b* are found by charting log *v* against log *I*. The current exhibits a precise correlation with the square root of the scanning velocity for *b* = 0.5, suggesting a diffusion-dominated process in which capacitance is derived from battery-type faradaic intercalation. The current and scan rate are closely correlated when *b* equals 1. This is an illustration of a capacitive response that combines surface pseudo-capacitance with EDLC capacitance. Surface active atoms participate in a fast faradaic charge transfer pathway that results in surface pseudo-capacitance. The *b* values corresponding to the anodic and cathodic peaks of MG-II//AC-ASC shown in Fig. 10(a) are 0.59 and 0.71, respectively, reflecting the redox characteristics of the charge storage process and double-layer contributions of MXene and graphene are responsible for the high diffusion capacitance behavior. The following relation was used to better quantify the capacitive contribution to the overall charge storage process in MG-II//AC-ASC supercapacitor^101^

16 *i*(*V*) = *k*_1_*v* + *k*_2_*v*^1/2^

**Fig. 10:**
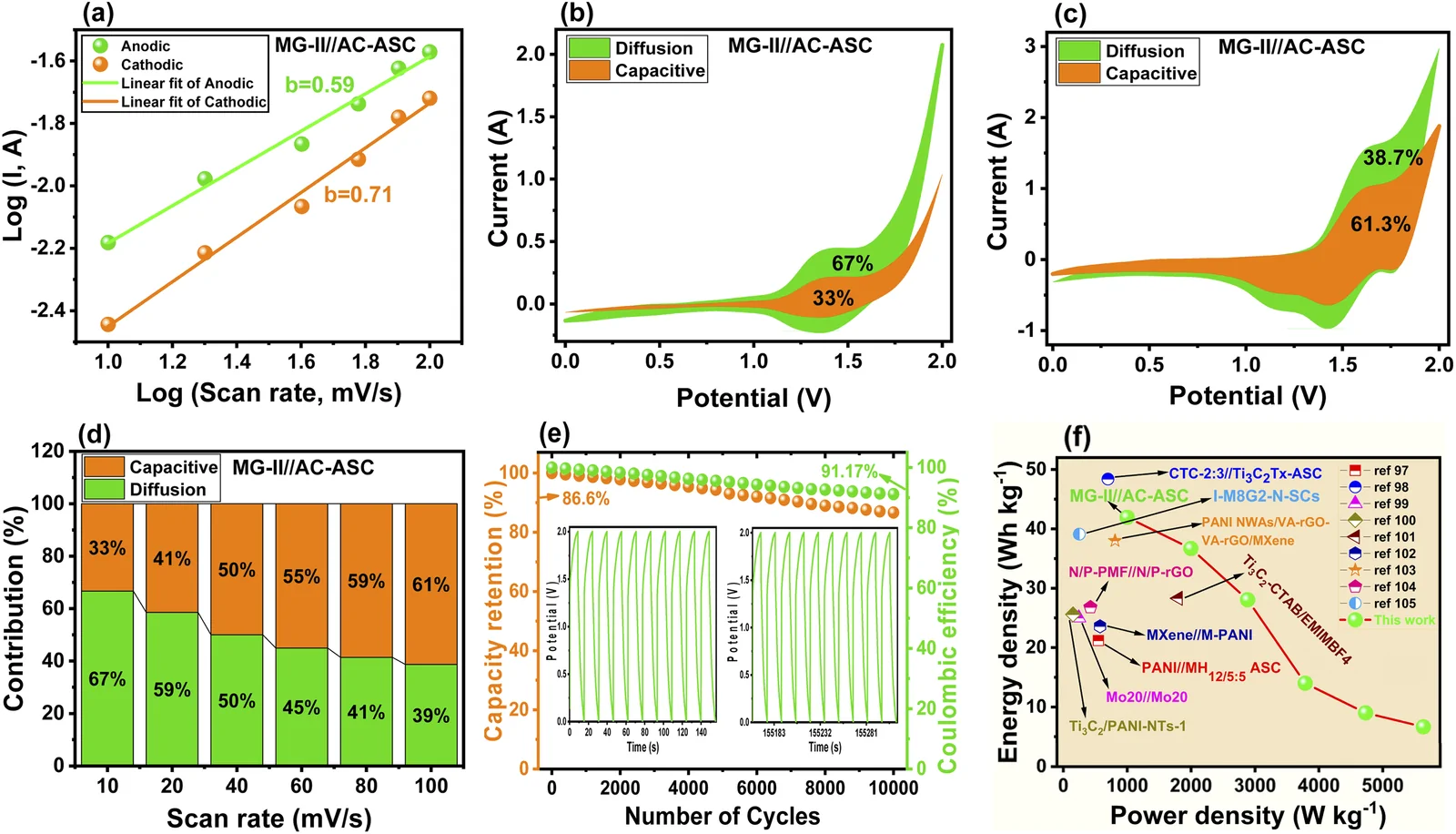
(a) *b* value plot for the fabricated device, (b and c) areal diffusion and capacitive influence graph at 10 and 100 mV s^−1^, respectively, (d) Scan rate dependence of capacitive and diffusion contributions to capacitance (e) % contribution of surface and diffusion controlled process at diverse scan rates (10–100 mV s^−1^), stability plot for 10 000 cycles at 5 A g^−1^ along with the inset of initial and final cycles, representing charge discharge profile, (f) Ragone plot with comparison of previous work.

The parameters *k*_1_*v* and *k*_2_*v*^0.5^ are associated with the influence of surface-controlled mechanisms and diffusion-controlled intercalation processes, respectively, where *i*(*V*) represent the current reaction at a specified voltage (*V*). By determining the *k*_1_ and *k*_2_ values, one can quantify the unique contributions from diffusion-controlled and capacitive mechanisms. The diffusion contribution of MG-II//AC-ASC is recorded at 67% of the total capacitance at a scan rate of 10 mV s^−1^, and it remains at 38.7% even for 100 mV s^−1^ scanning rate. Additionally, Fig. 10(b and c) clearly illustrate the diffusion capacitance contribution of MG-II//AC-ASC at scan rates of 10 mV s^−1^ and 100 mV s^−1^, respectively, is represented by the green shaded part in CV curves. These findings also show that, instead of being dominated by a surface-controlled mechanism, the charge storage in MG-II/AC-ASC is essentially pseudocapacitive at low scanning speed. Fig. 10(d) also shows rise in capacitive contribution with elevated scan rate, which is advantageous for high power applications.^102^ The cyclic stability of MG-II//AC-ASC electrode across 10 000 cycles as illustrated in Fig. 10(e), exhibiting good retention and steady coulombic efficiency as determined by eqn (9) and (10). The electrode retained 86.6% of its very first capacitance, demonstrating the MG-II//AC-ASC device's exceptional electrochemical reliability and endurance, which are pivotal for prolonged energy storage systems. The time it takes to attain a certain voltage at a constant current is monitored during each cycle, which consists of successive phases, the coulombic efficiency is around 91.17%. This indicates that there are very few losses while discharging and that nearly all of the charge delivered during the charging phase is recovered. This high coulombic efficiency is indicative of the system's electrochemical processes' exceptional reversibility. The voltage grew linearly during the charging and declined at a comparable rate during the discharging process, which is suggestive of optimal capacitive behavior.^103^ The charge–discharge curve's behavior is depicted in the inset of Fig. 10(e), with an emphasis on the initial ten cycles and the final ten. The material's reliable efficacy and effective charge storage are confirmed by the curves' almost symmetrical triangular configuration throughout the experiment.

The relationship between energy and power density of the MG-II//AC-ASC electrode as indicated by eqn (2) and (3), is represented in the Ragone figure. In Fig. 10(f), the energy and power densities of numerous MXene-based supercapacitor devices documented in the literature are juxtaposed. The ASC developed in this investigation demonstrates a maximum energy density of 41.95 Wh kg^−1^ at a power density of 1000 W kg^−1^, which is considerably higher than those found in earlier research.^104–112^ This provides more evidence that two dimensional MXene and graphene electrodes exhibiting comparable structural and electrochemical properties, might be surface engineered to create high-performance ASCs.

## 6. Conclusions

In summary, we examined the electronic and electrochemical properties of Ti_3_C_2_ MXene/graphene-composites. The addition of graphene changes the electronic properties of Ti_3_C_2_T_*x*_, specifically increasing the number of available electronic states at the Fermi level and causing charge transfer from Ti_3_C_2_T_*x*_ to graphene. Ti_3_C_2_T_*x*_/graphene composite was prepared using the basic one-step ultrasonication approach. XRD, Raman, and FTIR study revealed the formation of composites MG-I, MG-II and MG-III with different weight ratios 85 : 15, 70 : 30, and 55 : 45, with improved crystallinity, defective 2-dimensional architecture and the presence of remaining functional groups in the Ti_3_C_2_T_*x*_ sheets to the Ti_3_C_2_T_*x*_/graphene composite, respectively. DFT calculations on the simplified Ti_3_C_2_/graphene model indicate electronic redistribution and charge transfer from Ti_3_C_2_ toward graphene. FESEM images revealed that the surface morphology of the graphene flakes transformed the honeycomb-like structure with the incorporation of the Ti_3_C_2_T_*x*_ sheets. The charge/discharge curve indicated that the MG-II composite possesses a good specific capacitance of 366.3 F g^−1^ at 0.5 A g^−1^. The determined specific capacitance of the MG-II composite significantly surpasses that of graphene (124.8 F g^−1^), MXene (138.8 F g^−1^), and other composites MG-I (328.9 F g^−1^) and MG-III (247.5 F g^−1^) at an equivalent current density. The capacity retention of the MG-I, MG-II, and MG-III is about 80.31%, 85.39% and 78.03% respectively after 5000 cycles. The coulombic efficiency of the as-prepared composite with 30% filler (MG-II) showed a coulombic efficiency of 91.69% in the cycling stability as compared to pure materials graphene and Ti_3_C_2_T_*x*_. The electrochemical impedance measurements revealed improved interfacial charge transfer resistance of the MG-II composite. It is anticipated that the MG-II composite may serve as a viable material for high-efficiency supercapacitors. Furthermore, the asymmetric device operated at a voltage limit of 2 V and accomplished a specific capacitance of 75.5 F g^−1^ at a current density of 1 A g^−1^. Following 10 000 cycles, the device preserved the highest energy and power density of 41.95 Wh kg^−1^ and 1000 W kg^−1^, respectively, with remarkable coulombic efficiency and capacity retention of 91.17% and 86.6%. The study of Ti_3_C_2_T_*x*_/graphene composites suggest the way forward for the next generation for the fabrication of energy storage systems.

## Author contributions

Sakhi G. Sarwar & Muhammad Ahsan Iqbal: writing – review & editing, first-draft writing, methodology, data collection, software's, investigation and formal analysis, visualization, validation. Naveed Zafar Ali and Muhammad Arshad: conceptualization, visualization, validation, software, reviewing, and editing. Usama Shabbir and Qurat-ul-Ain: writing – review & editing, DFT calculations, software. M. Ashraf Gondal, TEM analysis and data curation, Serdar Altin and Shahzad Naseem investigation and visualization and writing – review.

## Conflicts of interest

The authors declare that they have no personal or financial conflicts that could have influenced the research described in this paper.

## Supplementary Material

RA-016-D6RA07651C-s001

## Data Availability

The data that support the findings of this study are available from the authors upon reasonable request. Supplementary information (SI): additional XRD patterns, SEM-EDX analyses, Raman and XPS spectra, CV curves, capacitive and diffusion contribution calculations based on Dunn's method, GCD profiles, and EIS spectra. See DOI: https://doi.org/10.1039/d6ra07651c.
