# Supplementary material for: Synergistic graphene–MXene heterostructures for enhanced energy storage in supercapacitors
Source: RSC Adv. 2026 Sep 29. Online ahead of print. doi: 10.1039/d6ra07651c (PMC13621509; doi:10.1039/d6ra07651c)
Supplement: Supplementary file 1 [file RA-OLF-D6RA07651C-s001.pdf]

Supplementary Information (SI) for RSC Advances.
This journal is © The Royal Society of Chemistry 2026
Supplementary Information (SI) for RSC Advances.
This journal is © The Royal Society of Chemistry

Supporting Information

Synergistic Graphene–MXene Heterostructures for Enhanced Energy Storage in Supercapacitors

Sakhi Ghulam Sarwar[1,2,3‡], Muhammad Ahsan Iqbal[2,4‡], Naveed Zafar Ali[2], Muhammad Arshad[5*], Usama Shabbir[6], Mohammad Ashraf Gondal[7], Qurat-ul-Ain[6], Shahzad Naseem[1], Serdar Altin[8*].

[1] Centre of Excellence in Solid-State Physics, University of the Punjab, Lahore, Pakistan.
[2] CoE-Physics, National Centre for Physics, Quaid-i-Azam University Campus, Islamabad, 44000, Pakistan.
[3] School of Material Science and Engineering, Beijing Institute of Technology, Beijing, China.
[4] Department of Physics, Division of Science and Technology, University of Education, Lahore, 54770, Pakistan.
[5*] Nanosciences and Technology Department, National Centre for Physics, Quaid-i-Azam University Campus, Islamabad, 44000, Pakistan.
[6] Department of Physics, Quaid-i-Azam University, Islamabad, 45320, Pakistan.
[7] Laser Research Group, Department of Physics & Interdisciplinary Research Center for Hydrogen Technologies and Carbon Management, King Fahd University of Petroleum and Minerals (KFUPM), Dhahran 31261, Saudi Arabia.
[8*] Battery Technology Application and Research Center & Physics Department, Inonu University, 44280, Malatya, Türkiye.
[9]Inonu University, Physics Department, 44280, Malatya, Türkiye.

Corresponding Author: Serdar ALTIN: serdar.altin@inonu.edu.tr , Tel. +90-536-9664468

Dr. Muhammad Arshad: arshad_pr2002@yahoo.com Tel. +92-3325084848

ǂ Authors contributed equally to this work.

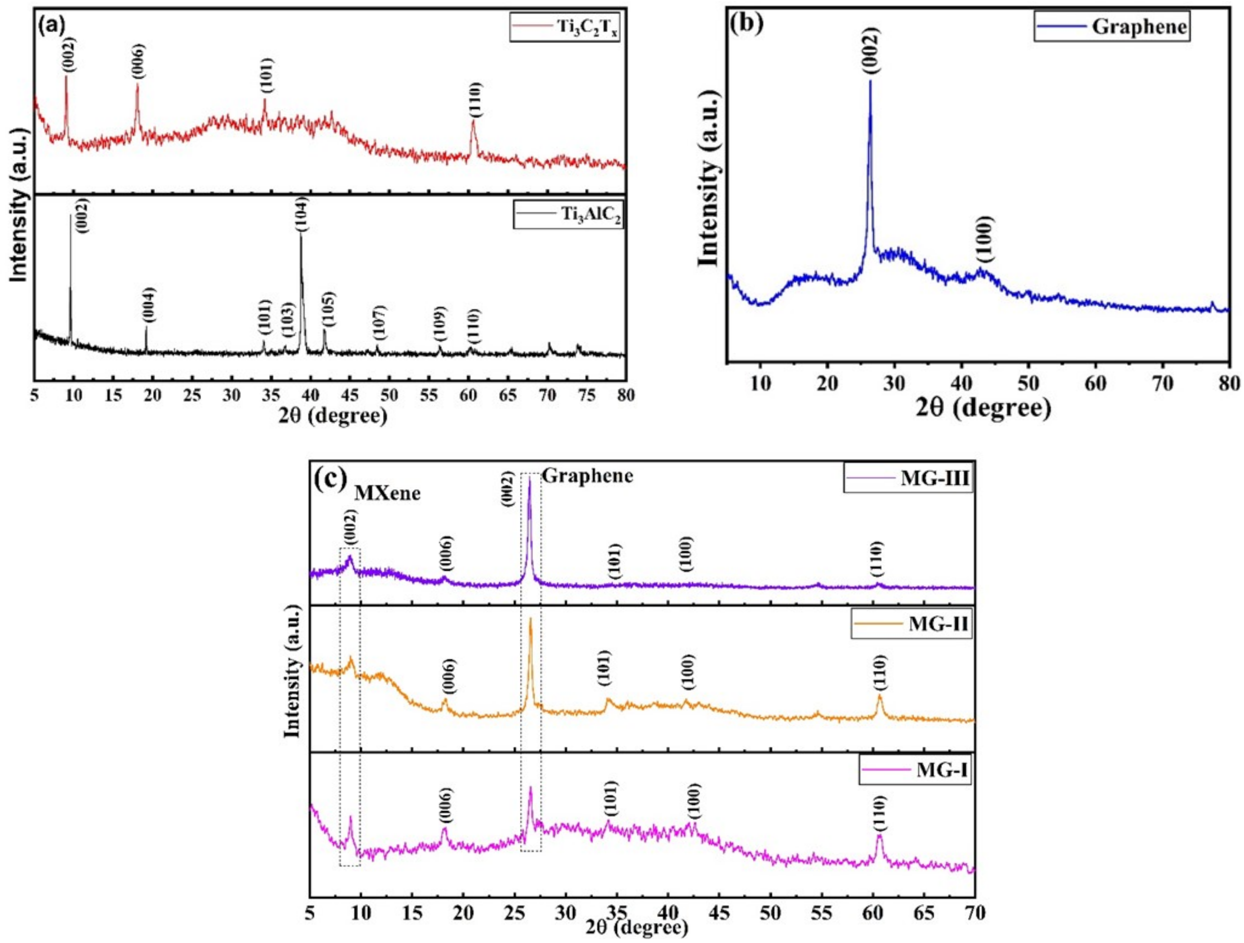

Fig. S1: XRD graph of (a) Ti_3AlC_2 and $Ti_3C_2T_x$ (b) graphene, (c) MG-I, MG-II, and MG-III composite.

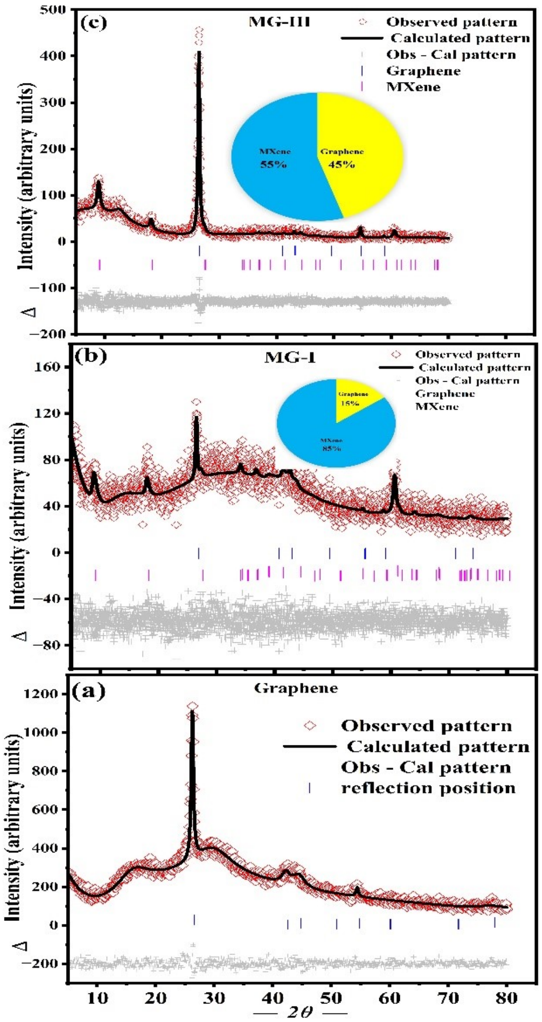

Fig. S2: Rietveld refinement of (a) Graphene, (b) MG-I, and (c) MG-III samples, respectively.

Table S1: Structural parameters for $Ti_3C_2T_x$ MXene/graphene composite samples obtained from Rietveld refinement.

**Samples Name**	**Hexagonal Phase (P63/mmc)**			**Vol. (Å^3)**	**Wt. % Rietveld**		**R_{wp}**	**R_{exp}**	**R_{Bragg}**
	**(a)**	**(b)**	**(c)**		**MXene**	**Graphene**			
$Ti_3C_2T_x$MXene	3.0326	3.0326	19.365	153.5254	100	0			2.3358
Graphene	2.4501	2.4501	6.6988	34.8264	0	100	7.619	6.665	0.6728
MG-I	5.5910	5.5910	25.912	190.8948	85	15	14.945	13.941	3.9009
MG-II	5.6050	5.6050	26.264	195.2659	70	30	13.977	12.663	4.1719
MG-III	5.5769	5.5769	26.105	192.3639	55	45	20.520	18.541	5.8760

Note: R_{wp}, R_{exp}, R_{Bragg} = R-factor

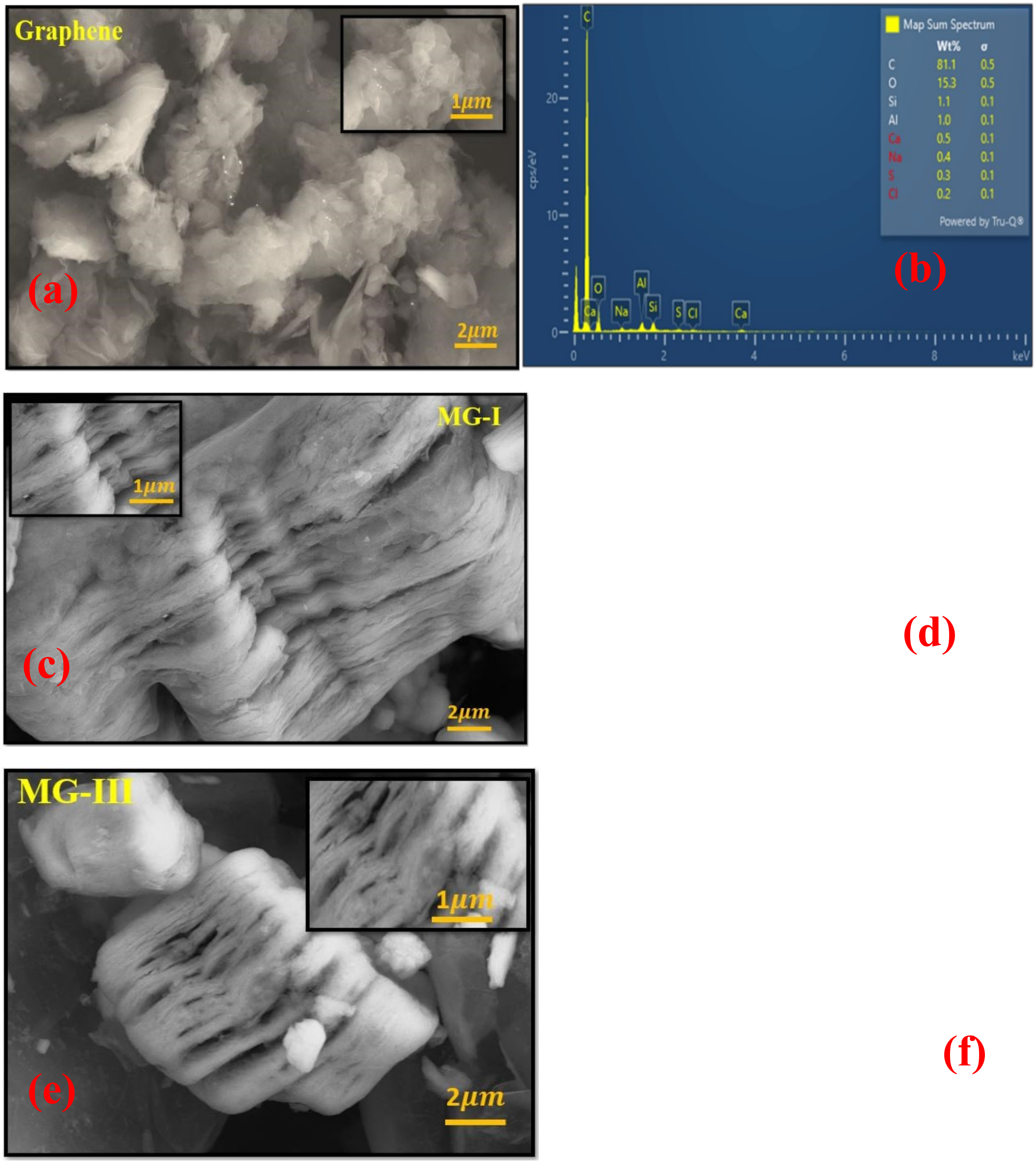

Fig. S3: SEM and Energy-dispersive spectra EDX spectrum of (a-b) graphene, (c-d) MG-I, and (e-f) MG-III composites.

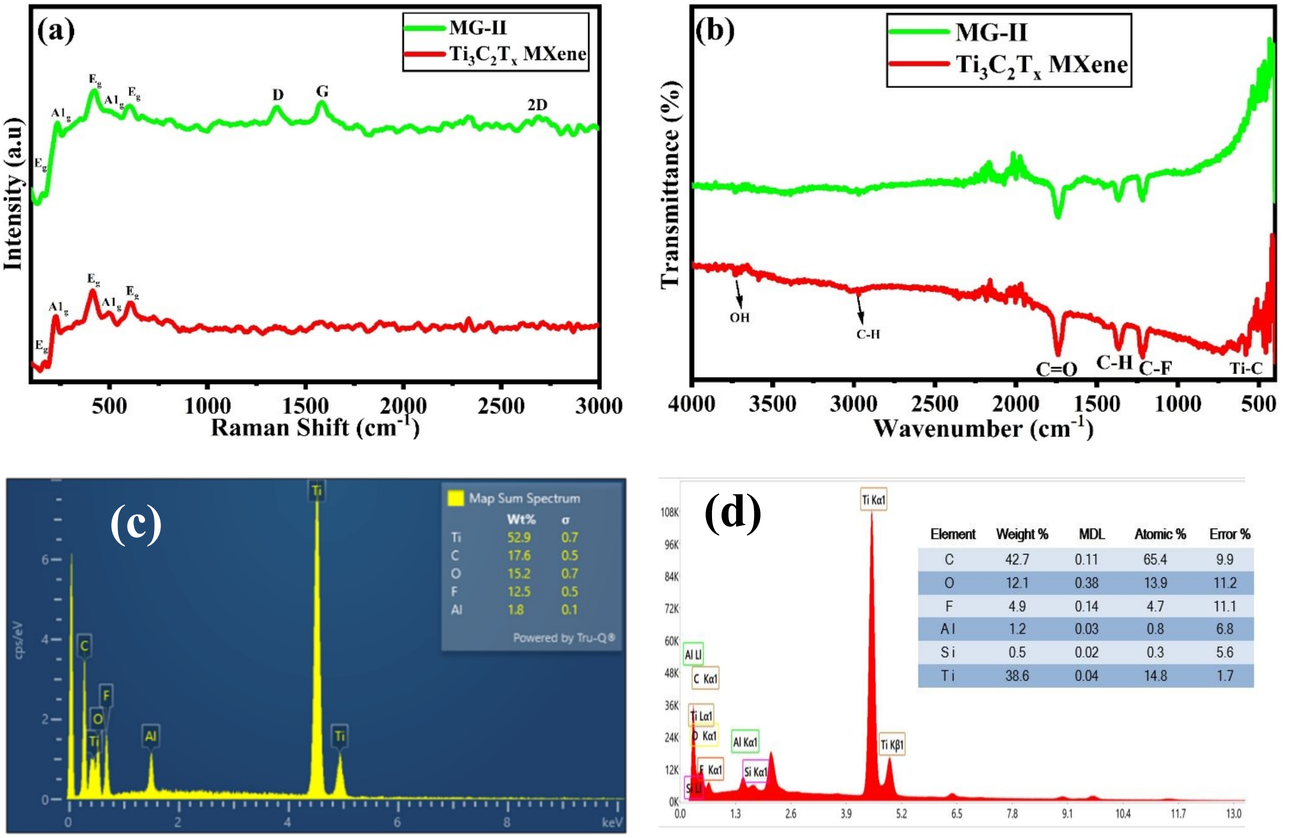

Fig. S4: (a) Raman spectra of $Ti_3C_2T_x$ MXene and MG-II composite. (b) FTIR spectrum of $Ti_3C_2T_x$ MXene, and MG-II composite. EDX images of (c) MXene (d) MG-II composites

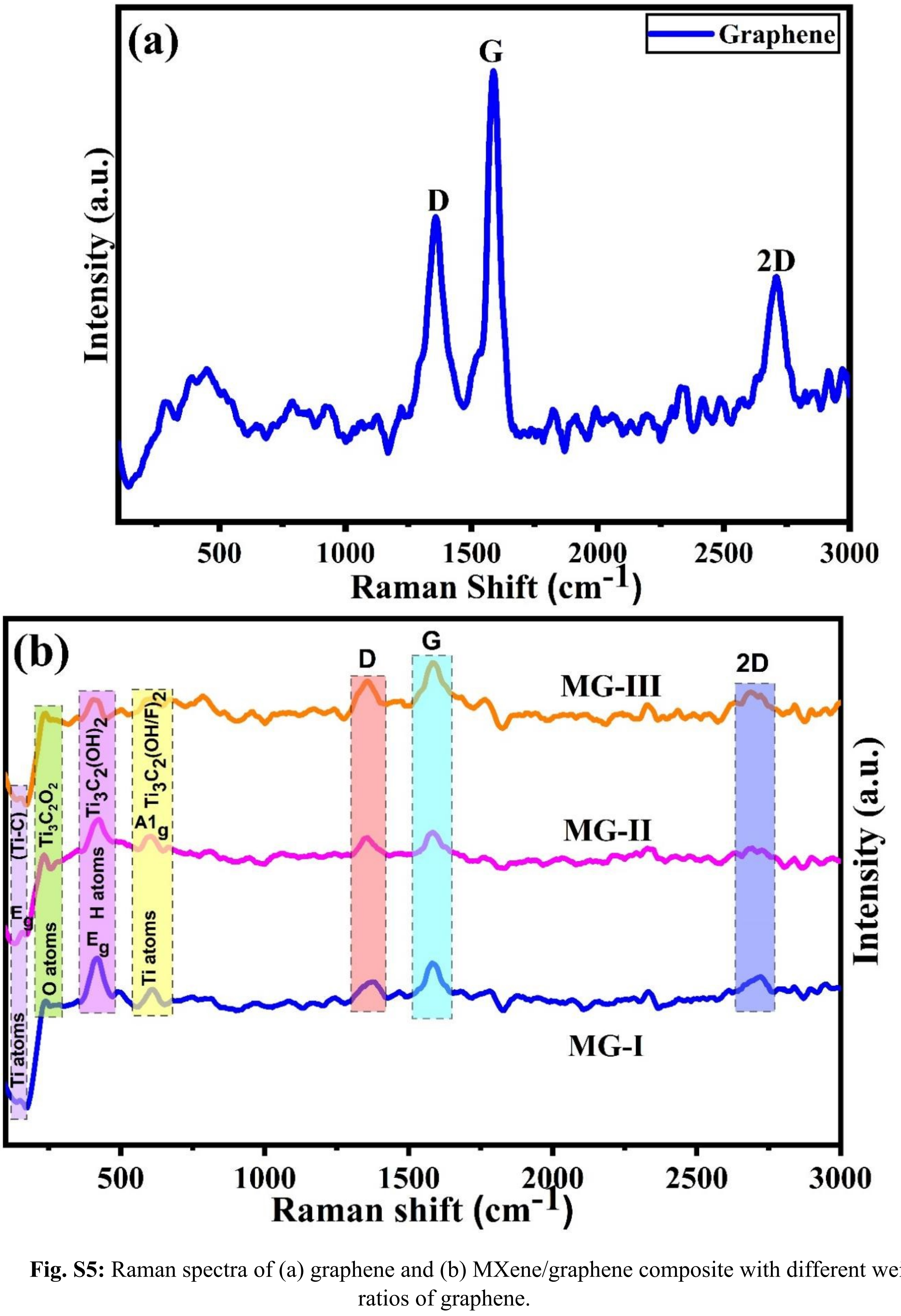

Fig. S5: Raman spectra of (a) graphene and (b) MXene/graphene composite with different weight ratios of graphene.

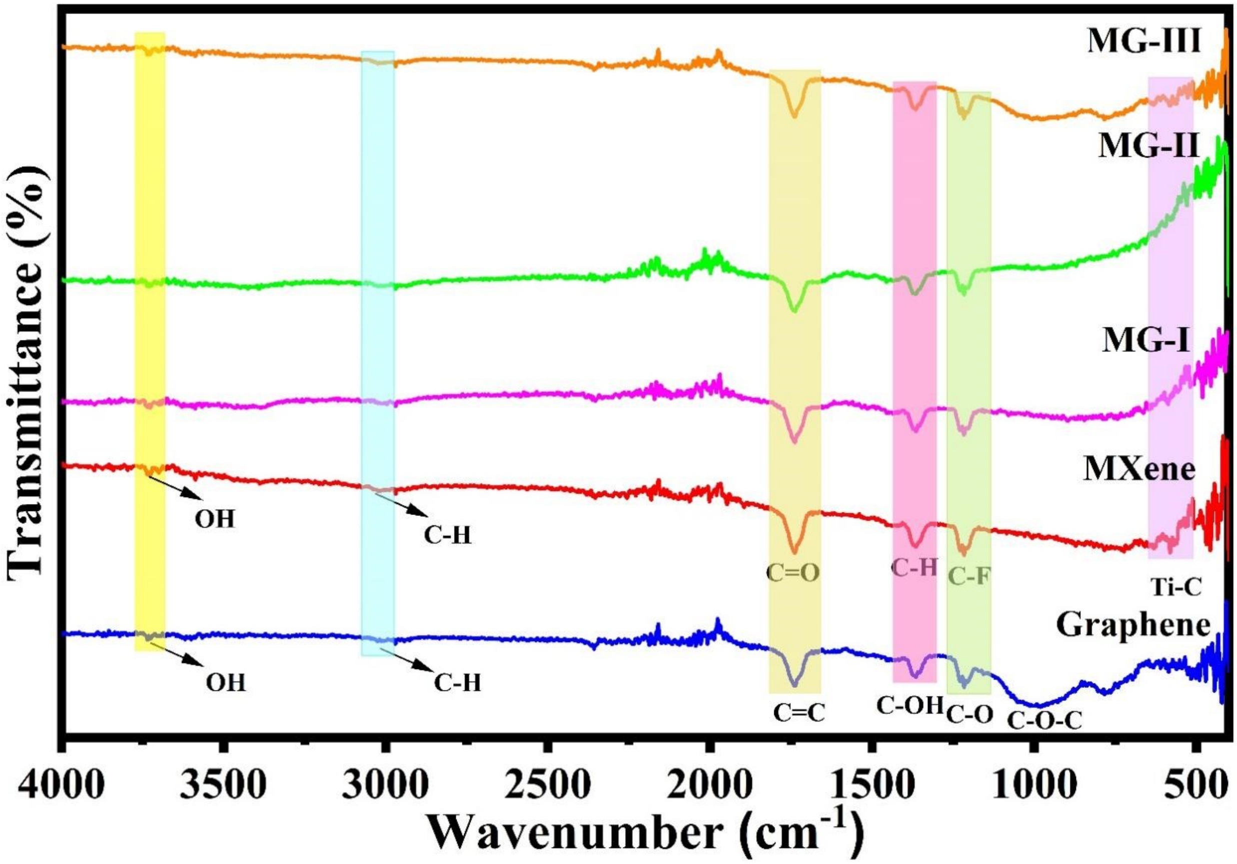

Fig. S6: FTIR spectrum of pure graphene, MXene, MG-I, MG-II, and MG-III composite.

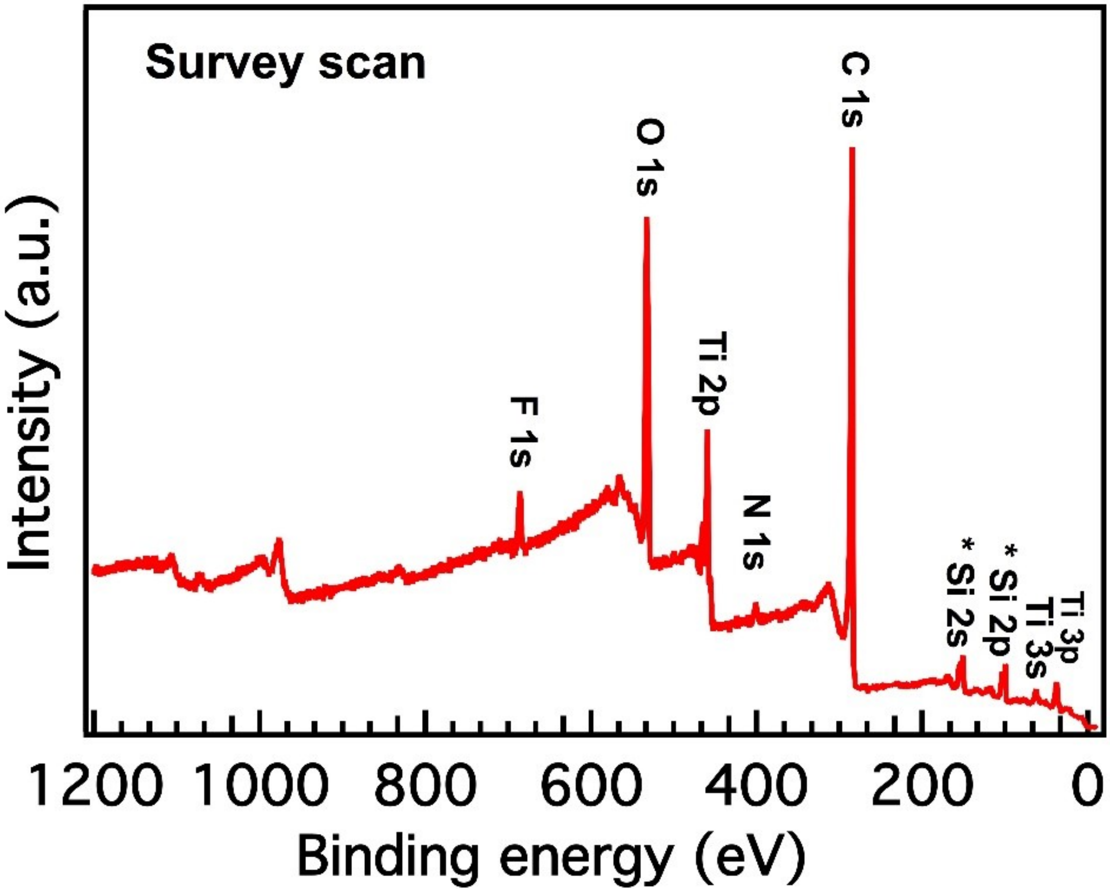

Fig. S7: The XPS survey data of MG-II composite.

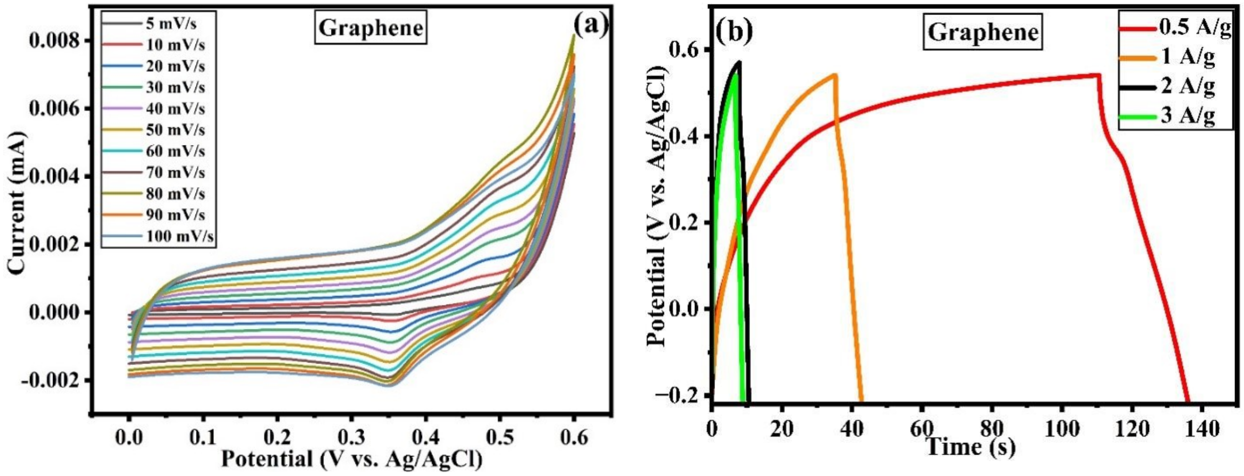

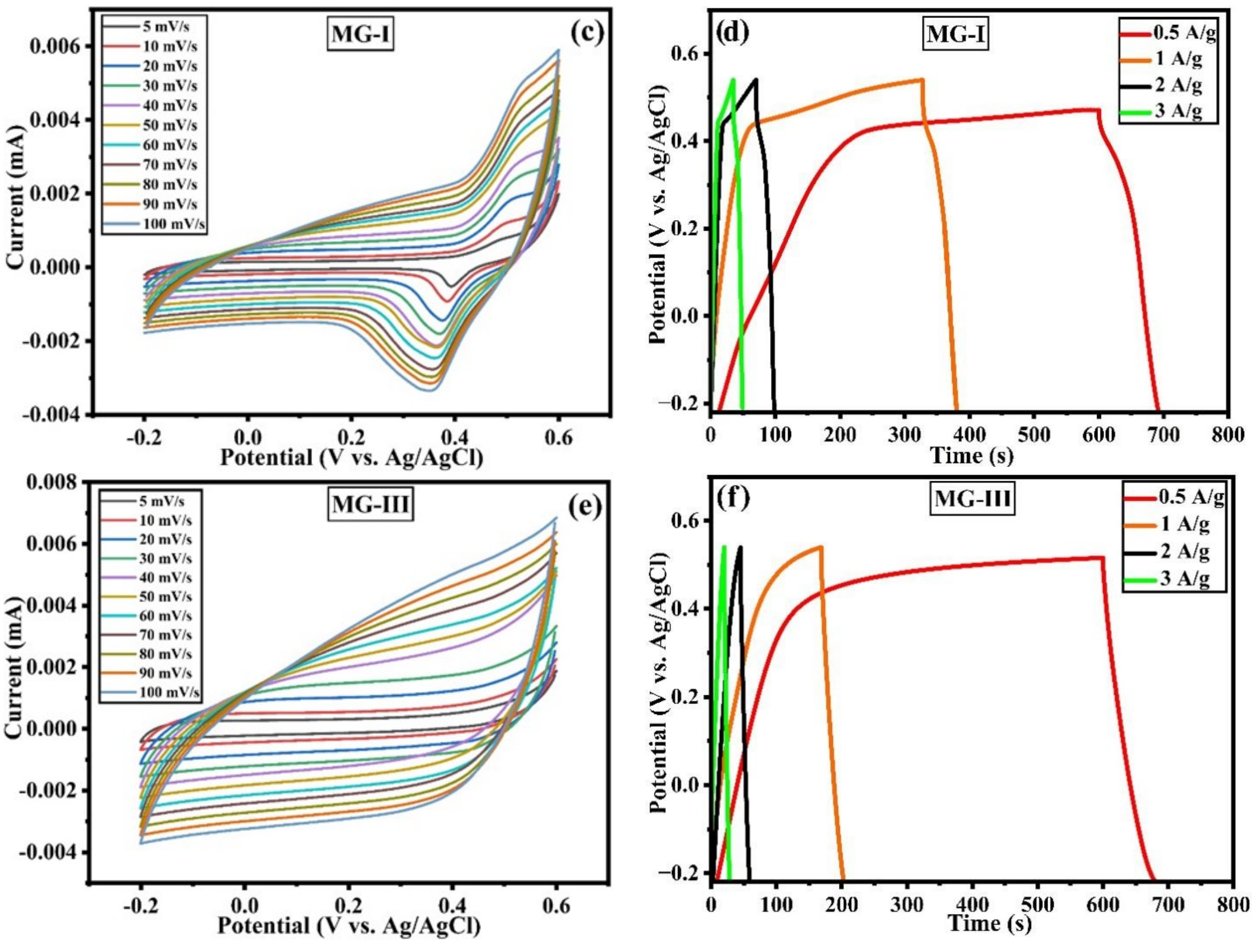

Fig. S8: CV curves at different scan rates and GCD graph with different current densities of (a,b) Graphene, (c,d) MG-I, and (e,f) MG-III respectively.

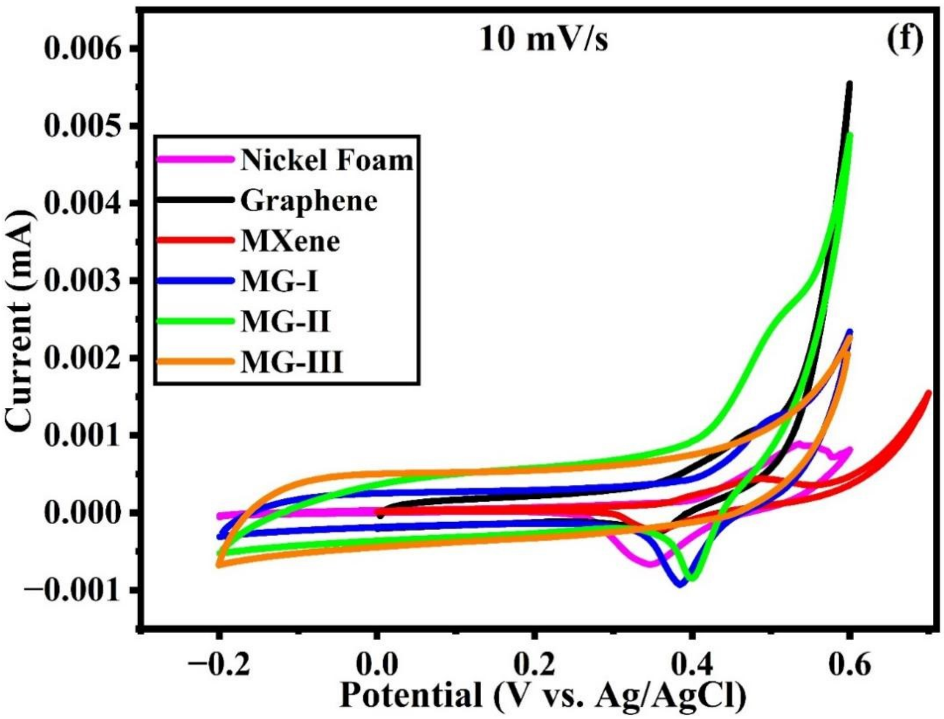

Fig. S9: Comparison of all materials CV curves with bare nickel foam at scan rate 10mV/s.

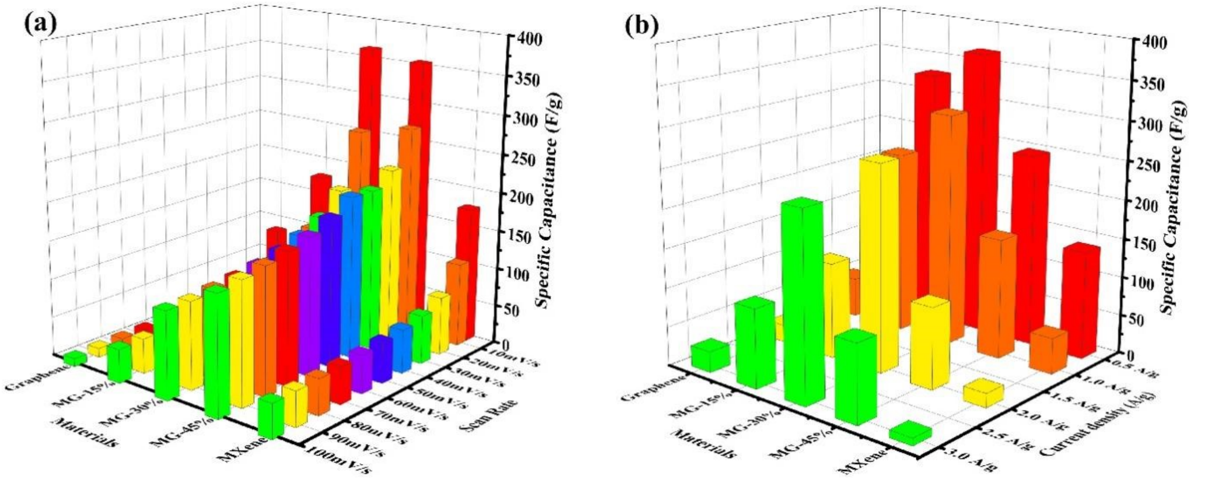

Fig. S10: A 3D graphical comparison graph b/w (a) capacitance vs. scan rate and (b) capacitance vs. current density.

Table S2: The specific capacitance of the bare and their composite materials was calculated from cyclic voltammetry (CV) curves at various scan rates.

**Materials**	**5 mV/s**	**10 mV/s**	**20 mV/s**	**30 mV/s**	**40 mV/s**	**50 mV/s**	**60 mV/s**	**70 mV/s**	**80 mV/s**	**90 mV/s**	**100 mV/s**
Graphene	82.9	41.1	20.4	17	14.9	13.9	13.3	12.8	12.3	11.6	10.5
MXene	176	108	74.8	62.5	56.1	52.2	49.2	47.4	45.9	44.6	43.5
MG-15%	266	201	155	137	125	120	112	106	102	97.9	94.3
MG-30%	544	384	277	236	214	199	188	180	174	167	164
MG-45%	354	272	226	208	209	191	178	170	163	157	152

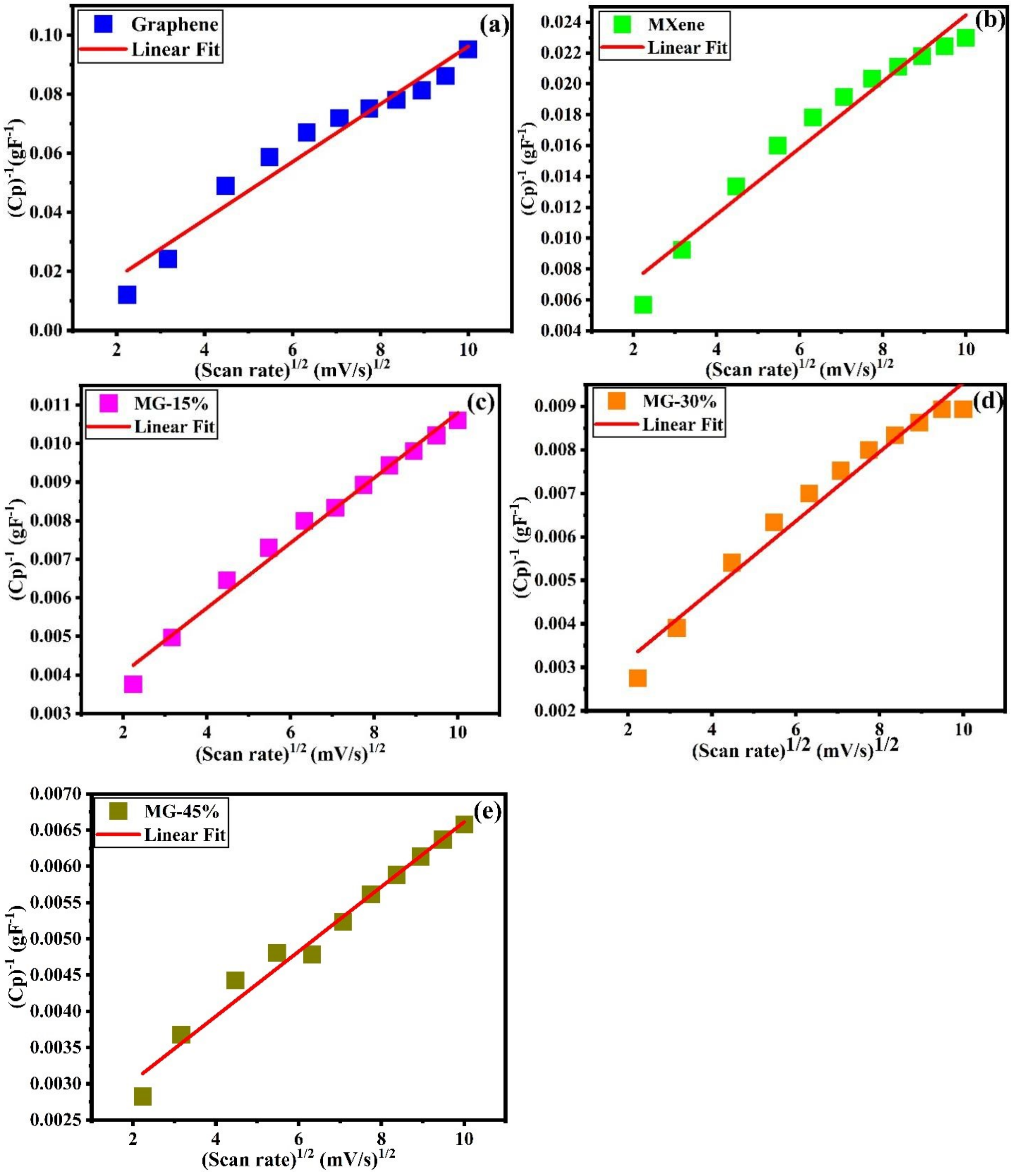

Graphene
Linear Fit
(a)
(Cp)^-1 (gF^-1)
(Scan rate)^1/2 (mV/s)^1/2
MXene
Linear Fit
(b)
(Cp)^-1 (gF^-1)
(Scan rate)^1/2 (mV/s)^1/2
MG-15%
Linear Fit
(c)
(Cp)^-1 (gF^-1)
(Scan rate)^1/2 (mV/s)^1/2
MG-30%
Linear Fit
(d)
(Cp)^-1 (gF^-1)
(Scan rate)^1/2 (mV/s)^1/2
MG-45%
Linear Fit
(e)
(Cp)^-1 (gF^-1)
(Scan rate)^1/2 (mV/s)^1/2

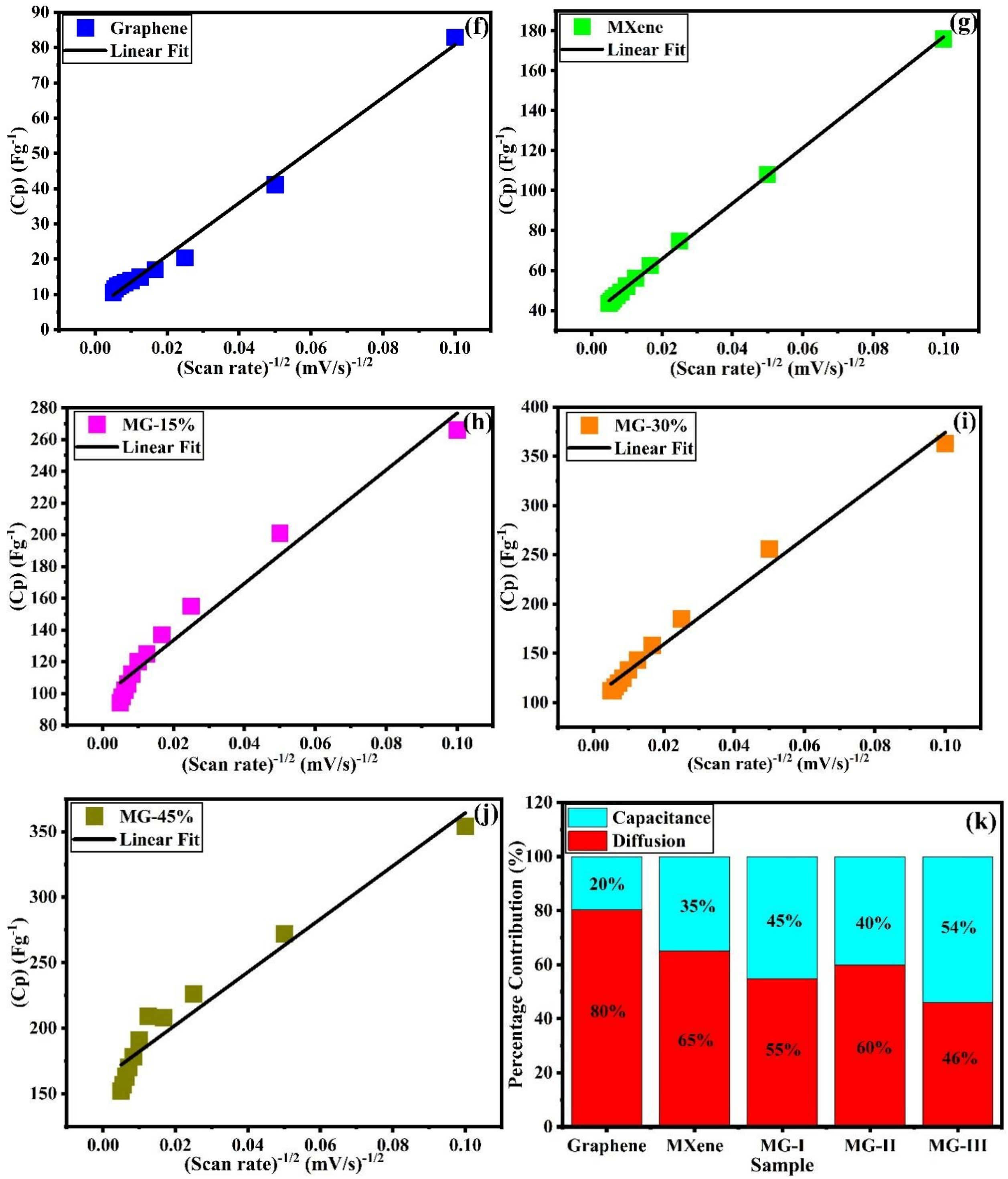

Fig. S11: (a-e) and (f-j) show linear fits of C^{-1} vs. $(\upsilon)^{1/2}$ and C vs. $(\upsilon)^{-1/2}$ for pure graphene, MXene, and its composites; (k) shows diffusive (PC) and capacitive (EDLC) charging based on Trasatti's method.

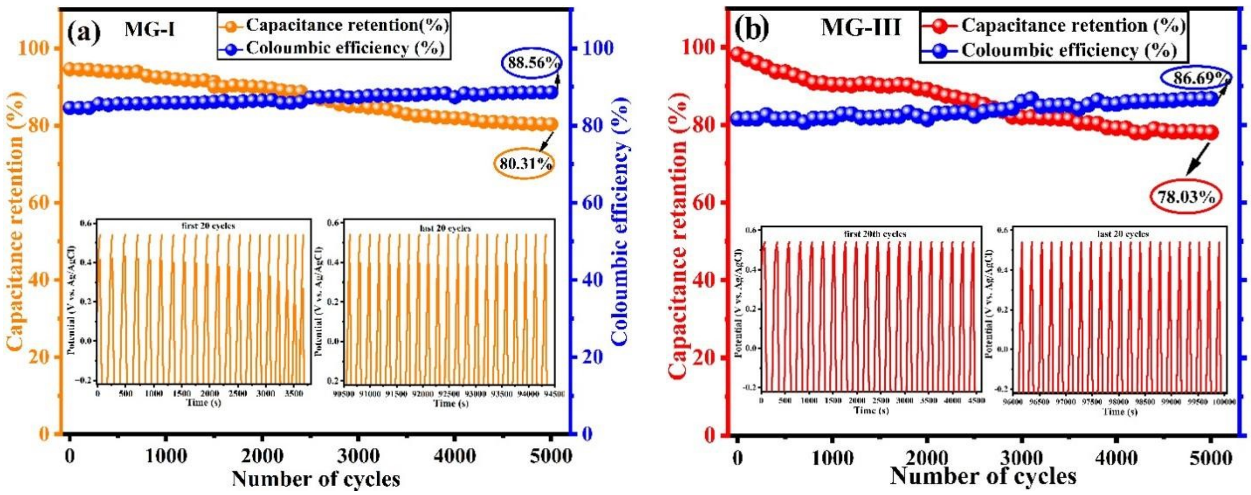

Fig. S12: A plot between the number of cycles vs capacitance retention and coulombic efficiency of (a) MG-I, (b) MG-III electrode materials at 1 A/g current density, (the inset graph shows the first and last 20th cycles of the charge-discharge).

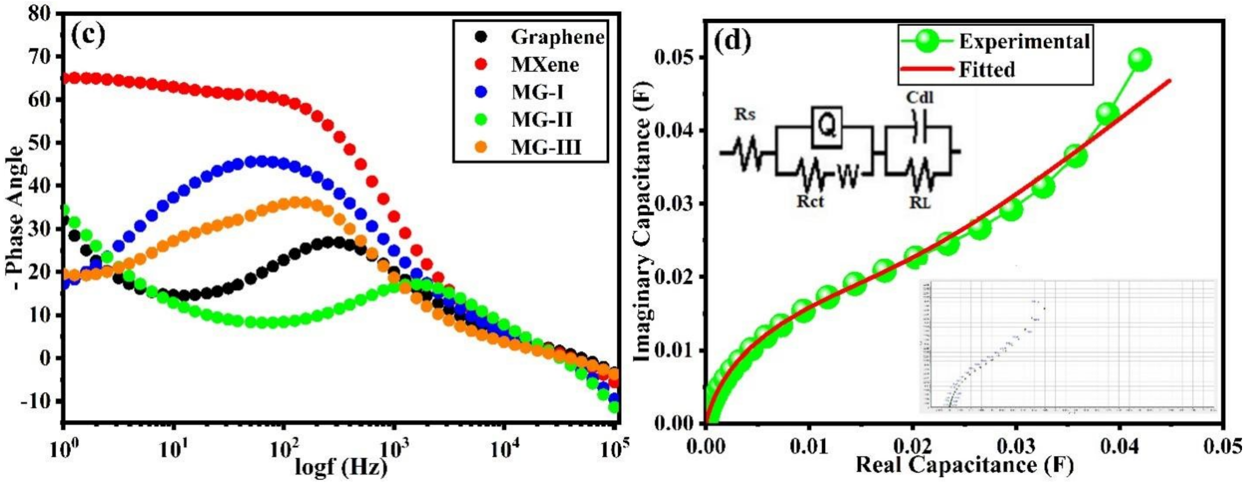

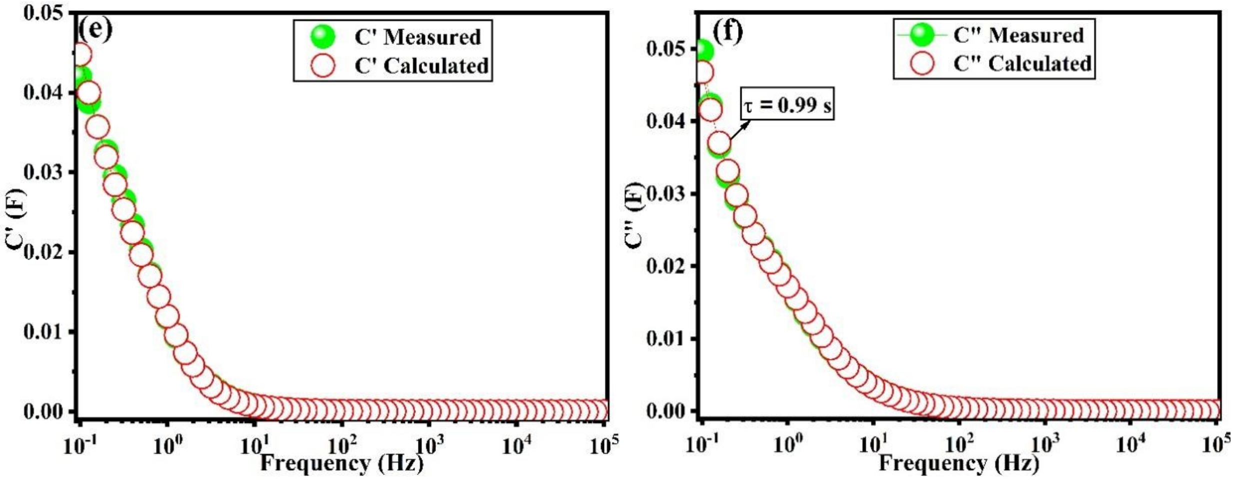

Fig. S13: (c) Bode plot, (d) real and imaginary capacitance plot; the inset is the equivalent circuit and fitted plot (e), (f).

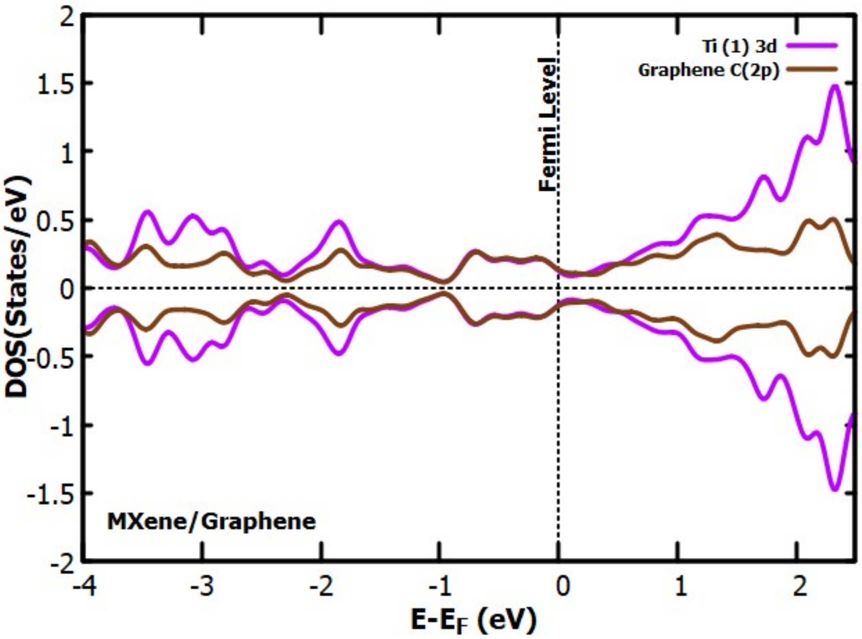

Fig. S14: Partial density of states of Ti 3d orbital and graphene (C) 2p. The bands are overlapping around the Fermi level.

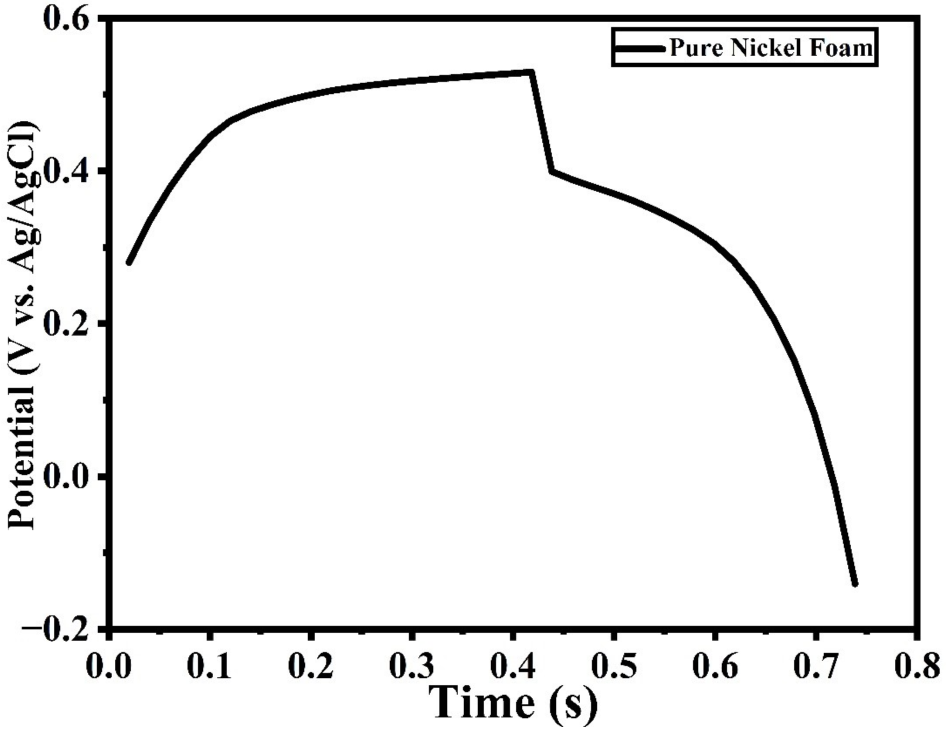

Fig. S15s: GCD graph of pure nickel foam.
